# Research and experimental verification of lightweight loader rims

**DOI:** 10.1038/s41598-024-62667-y

**Published:** 2024-05-23

**Authors:** Ma Long, Dong Zhikui, Liu Chunjiang, Gao Peng, Liang Ao, Hou Binfeng, Qi Xiangdong, Jiang Yunhong

**Affiliations:** 1 College of Mechanical Engineering, Yanshan University, Qinhuangdao, 066004 China; 2 Department of Applied Sciences, Northumbria University, Newcastle upon Tyne, NE1 8ST UK

**Keywords:** Construction machinery, Lightweight rims, Fatigue analysis, Response surface construction, Multiobjective optimization, Mechanical engineering, Materials science

## Abstract

The safety performance and structural stiffness of a rim, which is the main load-bearing structure of the loader during operation, influence the overall performance, stability, and braking capabilities of the machine. In the industry, researchers are currently pursuing lightweight and high-strength rims as a primary objective. A low weight not only enhances machinery fuel efficiency but also aligns with societal demands for sustainable development, energy conservation, and emission reduction. In this article, multiobjective optimization analysis on rims composed of three different materials is performed, and the relationships between various optimization parameters and target parameters are established using the results of response surface construction. Multiobjective genetic algorithms are utilized to derive various optimization plans, which are subsequently evaluated through static analysis, fatigue analysis, and weight loss analysis. The final optimization plan is determined based on the calculation results while considering production costs. Field tests are conducted on the optimized rims under various working conditions to verify the test results, evaluate the reliability of the finite element analysis results, and confirm the safety of the optimized rim.

## Introduction

Due to the rapid development of society, lightweight construction machinery has garnered widespread attention. The construction machinery examined in this article is a loader, and it operates under relatively complex conditions. As the main load-bearing component of the loader, the load on the wheel rim is irregular and random^1,2^. Therefore, after a long period of operation, the rim cracks due to the application of load, which affects the operation of the loader by the driver. Therefore, the strength and stiffness of the rim should be fully analysed when designing the rim to ensure the safety of the vehicle during operation. However, the life of the wheels should be guaranteed. If the fatigue lives of the wheels do not meet the usage requirements, frequent replacement of the wheels can lead to reduced economic benefits^3^. In short, the structural strength and service life of a wheel directly affect the stability, safety and braking performance of the loader during operation^4^. Currently, most loader rims are crafted from Q235 and can be categorized based on their structures into integral, two-piece, three-piece, and other rim types. The rim examined in this article is of the three-piece rim type, comprising a rim body, an outer rim, and a locking ring in its structure.

Domestic methods for designing rim structures mainly include analogous and trial-and-error methods, analytical methods, optimization design methods, and others^5^. In terms of reliability allocation, research has focused on optimal allocation^6–8^, fuzzy allocation^9,10^, redundant allocation^11–15^, and other related fields. Traditional wheel hub structural design primarily relies on the personal experience and past successful cases of engineers. Finite element simulation and bench testing are then used to adjust the dimensional parameters of specific areas of the wheel hub^16–19^. Li et al.^20^ employed numerical simulation to investigate the load-bearing performance of a wheel hub under impact tests. The authors predicted potentially dangerous failures in various parts of the wheel hub based on the stress distribution and validated the analysis results through impact tests. Wu et al.^21^ conducted orthogonal experiments to examine the stress distributions of wheel hubs with different shapes. These findings serve as a foundation for the design of wheel hub shapes. While the aforementioned research offers guidance for wheel hub design and optimization, these processes require extensive simulation and testing, making it a cumbersome, time-consuming, and labour-intensive process. Wang et al.^22,23^ performed a multiworking-condition optimization of the topology of a combined wheel hub, considering bending fatigue and radial fatigue. The scholars derived a wheel hub structure comprising a magnesium alloy rim and aluminium alloy spokes, which they optimized using a genetic algorithm. Pang^24^ and colleagues established a response surface model correlating various dimensions of spokes and rims with structural performance. The researchers conducted parameter optimization to achieve a lightweight material. The aforementioned research is primarily concentrated on the topology optimization of the wheel spokes and the size optimization of the rim. However, topological structure optimization and cross-sectional shape optimization for both rims and spokes are not considered. Furthermore, the current topology optimization of wheel spokes does not account for the rotational process of the rim. Hence, building upon existing research on the structural optimization of rims, further investigation into key technologies for rim structural optimization is imperative.

**Figure 1:**
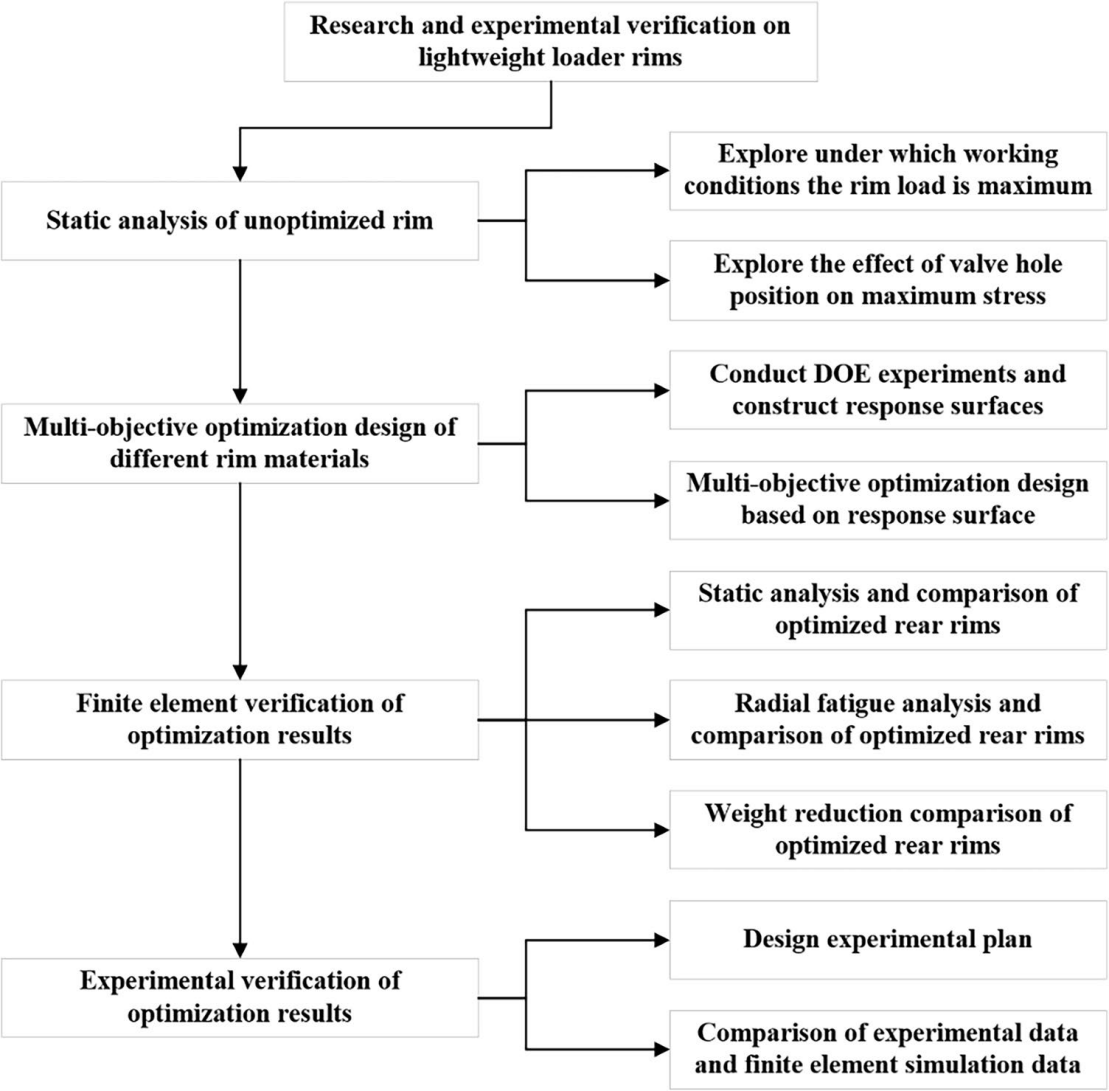
Key technology flow chart.

In this article, a static analysis of the existing wheel rim is conducted to explore the effects of rim load and different valve hole positions on the maximum stresses under different working conditions. A DOE (Design of Experiments) experiment is conducted on the rim with the valve hole positioned upwards when the rear wheel is lifted off the ground. A response surface is constructed based on the above static analysis results, and a multiobjective optimization design is constructed based on the response surface. Static analysis, radial fatigue analysis and weight loss analysis are conducted on the optimized rear rim. Finally, experimental verification is carried out to increase the credibility of the finite element analysis results. The key technology flow chart of this article is shown in Figure 1.

## Researching the overall structure and simulation model of the wheel rim

### Investigating rim size and material parameters

Due to the numerous rim size variables, we initially process the rim size parameters, analyse their impacts on the rim size parameter alterations, and subsequently select the optimal rim size.

**Figure 2:**
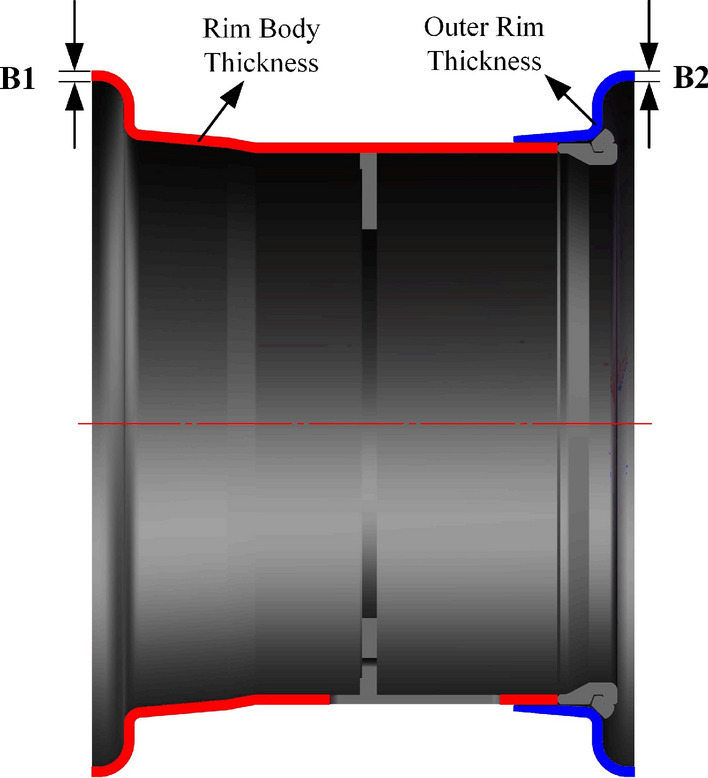
Schematic diagram of the Rim structure optimization parameters.

To maintain the unchanged assembly relationships among the rim, tire, and drive axle and to ensure the normal functionality of the valve hole during tire inflation, it is decided, after analysis, to perform optimization by adjusting the rim body wall thickness and outer rim wall thickness while keeping the other dimensions unchanged, as shown in Fig. 2.

Currently, Q235 is the primary material used in engineering machinery rims. However, in this paper, the Q355 and 540CL materials are chosen as the optimal materials for the rims.

Considering different physical parameters, such as the yield strengths and tensile strengths of different materials, this multiobjective optimization process entails distinct optimization ranges for the parameters based on the material, as depicted in Table 1. The optimization targets include the maximum stress (S) and rim quality (M).

**Table 1:** Parameterized range of wheel rim model dimensions.

Dimensional code	Material	Component	Initial value	Optimization upper limit	Optimization lower limit
B1	Q235	Wheel rim thickness	11.5 mm	11.5 mm	8 mm
	Q355		11.5 mm	11.5 mm	7 mm
	540CL		11.5 mm	11.5 mm	6 mm
B2	Q235	Outer rim thickness	11.5 mm	11.5 mm	8 mm
	Q355		11.5 mm	11.5 mm	7 mm
	540CL		11.5 mm	11.5 mm	6 mm

### Investigating the physical parameters of rim materials

The steel plates needed for wheel rim production are processed into bar-shaped tensile specimens using wire cutting, turning, and other methods. Tensile testing is conducted using a Zwick-Z100 material universal testing machine at a rate of 1 mm/min. The specimens are stretched to complete the fracture process to collect stress–strain data.

**Figure 3:**
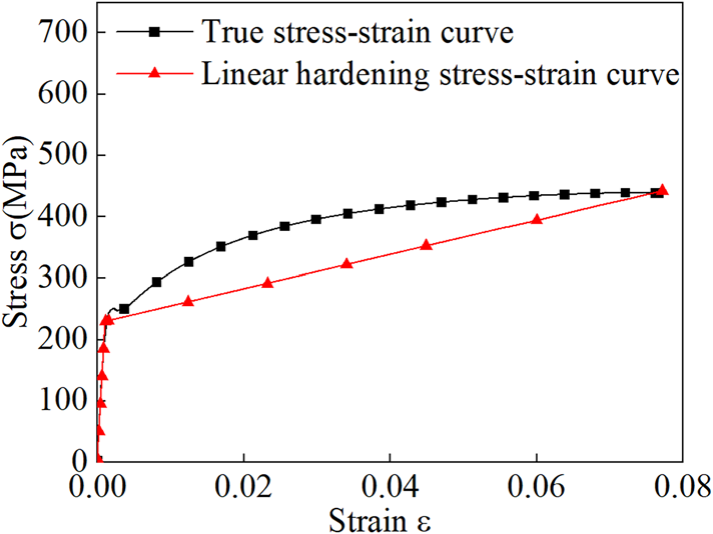
Bilinear model for the Q235 material.

**Figure 4:**
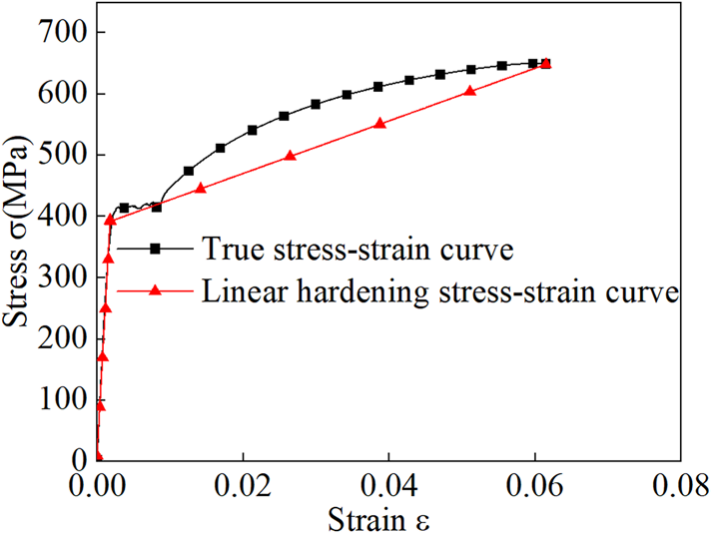
Bilinear model for the Q355 material.

**Figure 5:**
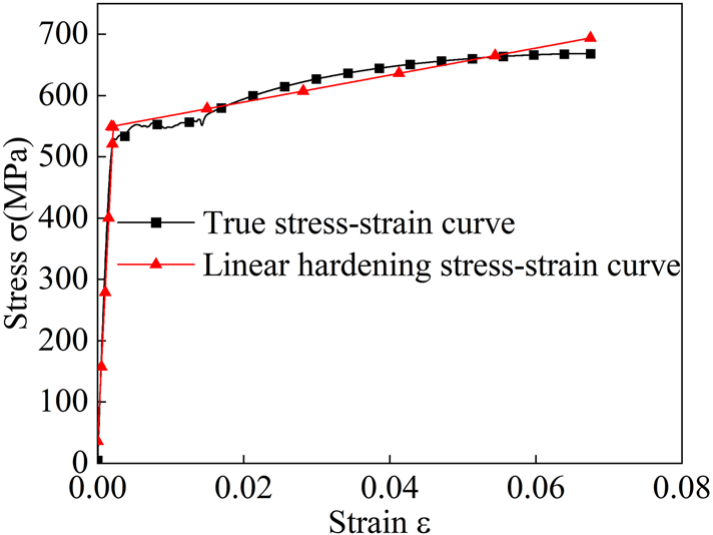
Bilinear model for the 540CL material.

The stress-strain data acquired from the test are in the form of engineering stress-strain data, requiring conversion into true stress-strain data. The bilinear models for the three materials are illustrated in Figs. 3, 4 and 5, while the specific material parameters are detailed in Table 2.

**Table 2:** Physical parameters of the three steel materials.

Experimental material properties	Q235 (wheel rim)	Q355 (wheel rim)	540CL (wheel rim)
Density ${ \rho (kg/m^3) }$	7850	7850	7850
Young’s modulus E (MPa)	2.09E+05	2.10E+05	2.32E+05
Poisson’s ratio ${ \gamma }$	0.28	0.3	0.3
Yield strength (MPa)	240	400	520
Tensile strength Rm (MPa)	415	551	660
Tangent modulus (MPa)	2.49+E3	2.90+E3	2.50+E3

### Processing the finite element model of the wheel rim

The rim examined in this article is a three-piece rim. Owing to the intricate structure of the rim, which includes small features such as fillets, chamfers, and gaps, meshing the finite element is challenging, leading to the difficulties of convergence in calculations. Hence, three-dimensional modelling software is utilized to simplify these complex microstructures.

The grid sizes are set to 50 mm, 20 mm, 10 mm, and 5 mm. The errors of the numerical solutions for the 10 mm case and the 5 mm case are both 6$\%$, and the grid size is selected to be 10 mm.

**Figure 6:**
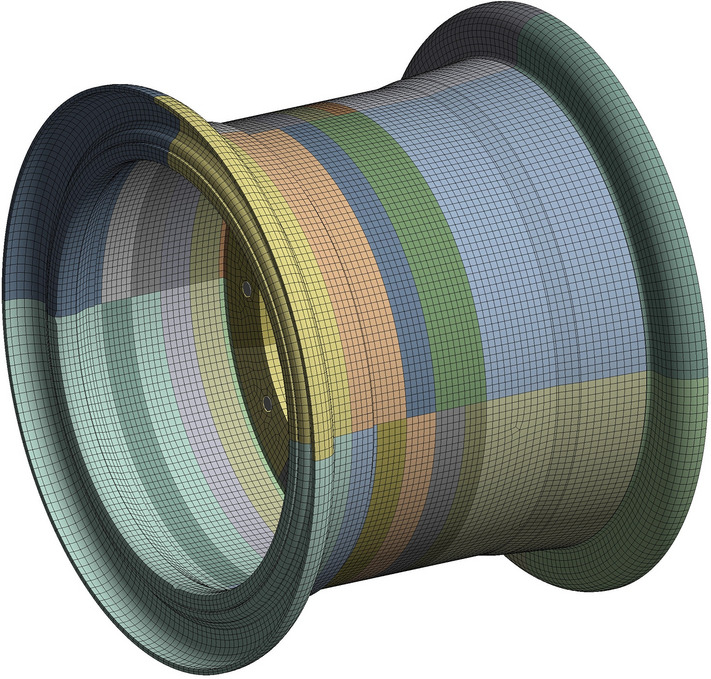
Finite element mesh model of the wheel rim.

**Figure 7:**
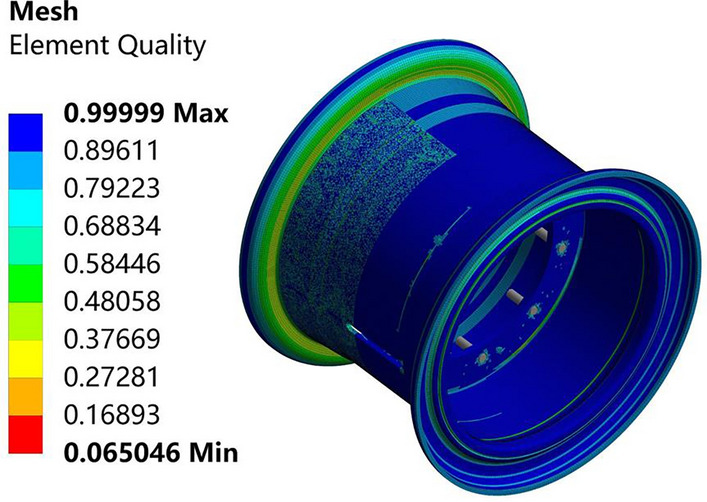
Rim mesh quality diagram.

After comprehensive consideration, to ensure calculation accuracy and efficiency, the rim is divided into hexahedral meshes. The mesh size is 10 mm, the number of nodes is 173,100, and the number of meshes is 29,213. The average element quality is 0.84624, the average orthogonal quality is 0.82463, the average skewness is 0.22362, and the average aspect ratio is 1.8473. The mesh quality is considered good in this study. Figure 6 shows the finite element mesh model of the rim, and Fig. 7 shows the rim mesh quality diagram.

### Static analysis of the wheel rim

The construction of the response surface relies on the analysis of static data. By modifying the rim wall thickness and rim material, the maximum stress of the rim under identical working conditions can be altered.

Before conducting static analysis on the rim, the following boundary conditions must first be established in the finite element simulation software:

- The gravitational acceleration is set to the default value of − 9806.6 mm/$\hbox {s}^{2}$, with the direction being − Y.
- A pretightening force of 57,390 N is applied to the bolts on the wheel spokes and drive axle.
- The friction coefficient between the ground and the tire is set to 0.3 and that between the rim and the tire is set to 0.9. The normal Lagrangian method is employed to enforce nonpenetrating contacts and designate it as the convergence target.
- The remote displacement constraint is utilized to restrict the remaining 5 degrees of freedom of the spoke centre plane except for the Y direction, ensuring that the rim and tire can only experience displacement in the Y direction while constraining all 6 degrees of freedom of the ground.
- An air pressure of 0.4 MPa is applied perpendicular to the surfaces of the tire inner wall and rim body.
- The distal force is applied to simulate the force transmitted by the drive axle through the wheel spokes in the − Y direction.
- This simulation comprises two load steps. The first load step involves tire inflation, while the second load step applies force. The calculation time for each load step is set to 1 s. Large deformation settings are disabled. The minimum substep for each load step is set to 10, with an initial substep of 10 and a maximum substep of 100.

**Figure 8:**
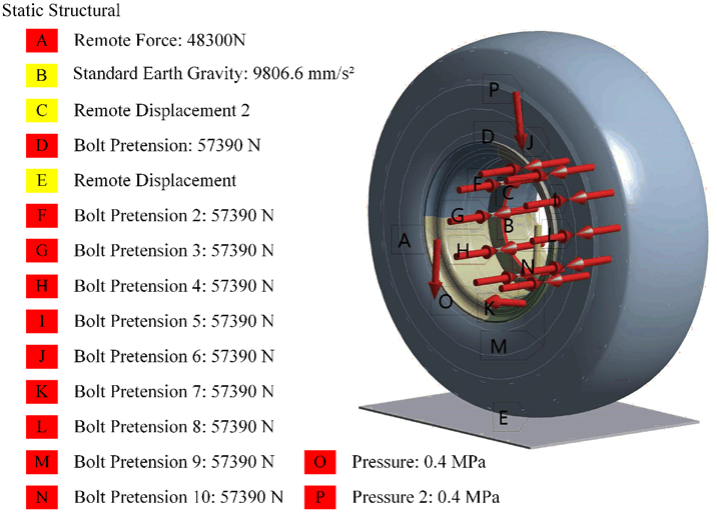
Overall boundary conditions for wheel rim statics.

The constraints and load application of the entire simulation are shown in Fig. 8.

The load on the rim is related to the working conditions of the loader. The forces on the front and rear wheels are different under distinct working conditions^25,26^. In this article, a mechanical analysis on two common working conditions of the loader is conducted and mechanical models are established, namely, the mechanical model of the case in which the four wheels are on the ground and that of the case in which the rear wheels are tilted and only the front wheels are stressed during shovelling. Since only the working conditions of the loader on a smooth road are studied and because its load is symmetric, this component is simplified into a two-dimensional force model.

The rim studied in this article is a rim on a medium-sized loader of a company. The rated maximum load of the loader is 5 t. The specific performance parameters are shown in Table 3 below.

**Table 3:** Loader performance parameters.

Performance parameters	Value
Rated bucket capacity (m^3^)	3
Rated maximum load (t)	5
Rated power (kw)	170
Unloading height (mm)	3420
Tipping load (t)	13
Body weight (t)	15.5
Bucket weight (t)	2
Maximum rising power (t)	17.5 ± 5
Largest traction (t)	16.5

**Figure 9:**
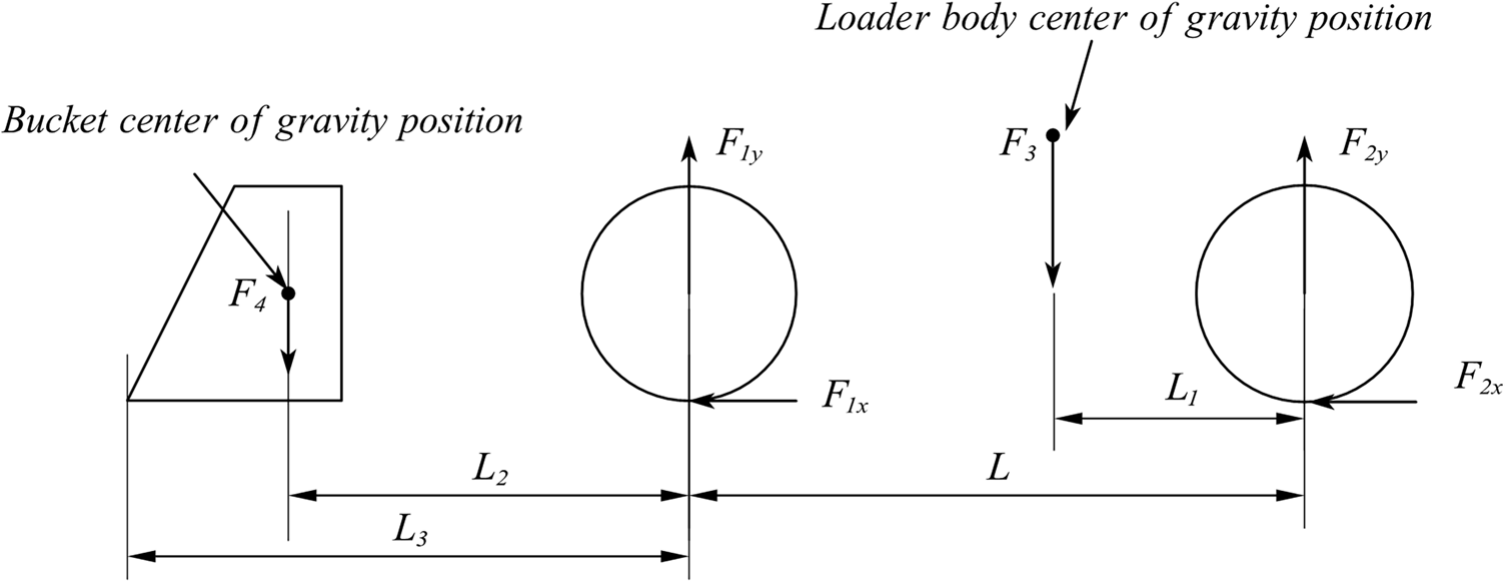
Four-wheel landing mechanics model.

The stress model of the four-wheel landing condition of the loader is shown in Fig. 9:

A force analysis in the vertical direction yields the following expression:

1 $$\begin{aligned} \sum {F_y} = {F_{{\textrm{1y}}}} + {F_{{\textrm{2y}}}} - {F_\mathrm{{3}}} - {F_\mathrm{{4}}} = 0 \end{aligned}$$

where F_1__y_, ground reaction force on the front wheel (N). F_2__y_, ground reaction force on the rear wheel (N). F_3_, loader body weight (N). F_4_, bucket and shovel material weight (N).

the moment at which the front wheel contacts the ground is considered:

2 $$\begin{aligned} {L_2} \times {F_\mathrm{{4}}} + L \times {F_{{\textrm{2y}}}} = {F_\mathrm{{3}}} \times \left( {L - {L_1}} \right) \end{aligned}$$

where L_1_, distance between the rear wheels and the loader centre of gravity (mm). L_2_, distance between the front wheel and bucket centre of gravity (mm). L, distance before the front and rear wheels (mm).

Equations (1) and (2) are solved simultaneously to obtain the mathematical expression of F_1__y_ and F_2__y_:

3 $$\begin{aligned} \left\{ \begin{array}{l} {F_{{\textrm{1y}}}} = \left( {1 + \frac{{{L_2}}}{L}} \right) {F_\mathrm{{4}}} + \left( {\frac{{{L_1}}}{L}} \right) {F_\mathrm{{3}}}\\ {F_{{\textrm{2}}y}} = \left( {1 - \frac{{{L_1}}}{L}} \right) {F_\mathrm{{3}}} - \left( {\frac{{{L_2}}}{L}} \right) {F_\mathrm{{4}}} \end{array} \right. \end{aligned}$$

**Figure 10:**
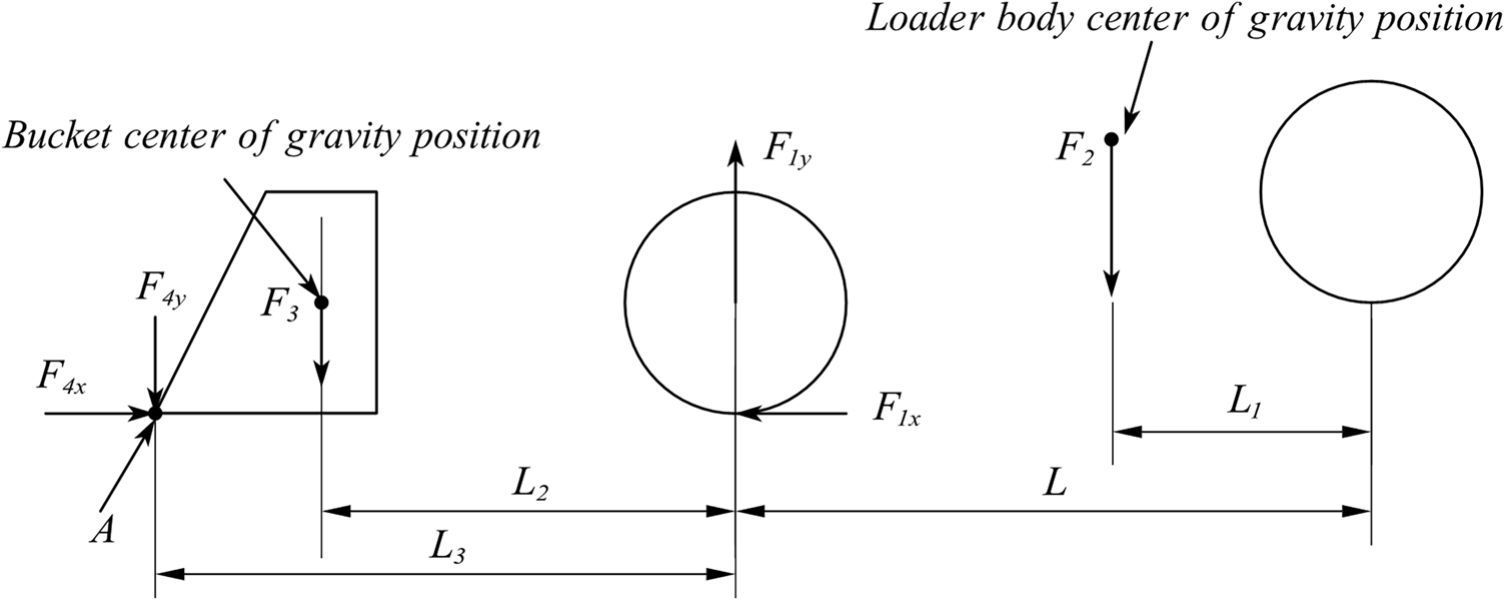
Rear wheel lift mechanics model.

The force model of the rear wheel lifting condition of the loader is shown in Fig. 10:

A force analysis in the vertical direction yields the following expression:

4 $$\begin{aligned} \sum {F_y} = {F_{{\textrm{4y}}}} + {F_3} + {F_2} - {F_{{\textrm{1}}y}} = 0 \end{aligned}$$

where F_4__y_, rising resistance of materials to the bucket (N). F_1__y_, support force of the ground facing the front wheel (N). F_3_, bucket weight (N). F_2_, loader body weight (N).

The moment at point A where the bucket contacts the ground is obtained:

5 $$\begin{aligned} {F_3} \times \left( {{L_3} - {L_2}} \right) + {F_2} \times \left( {L - {L_1} + {L_3}} \right) = {F_{{\textrm{1}}y}} \times {L_3} \end{aligned}$$

where L_1_, distance between the rear wheels and the loader centre of gravity (mm). L_2_, distance between the front wheel and bucket centre of gravity (mm). L_3_, distance from the front wheel to the bucket front (mm). L, distance before the front and rear wheels (mm).

By solving Eqs. (4) and (5), the following mathematical expressions of F1y and F4y can be obtained:

6 $$\begin{aligned} \left\{ \begin{array}{l} {F_{{\textrm{1}}y}} = \left( {1 + \frac{{L - {L_1}}}{{{L_3}}}} \right) {F_2} + \left( {1 - \frac{{{L_2}}}{{{L_3}}}} \right) {F_3}\\ {F_{4y}} = \left( {\frac{{L - {L_1}}}{{{L_3}}}} \right) {F_2} + \frac{{{L_2}}}{{{L_3}}}{F_3} \end{array} \right. \end{aligned}$$

By bringing different load sizes into the stress model of the corresponding working conditions, the corresponding force sizes of the front and rear wheels are obtained, as shown in Table 4. The data in the table clearly show that when the rear wheel is lifted off the ground, the rim load is the largest.

**Table 4:** Load magnitude in different conditions.

Operating condition	Load magnitude	Vehicle weight	Bucket weight	Front wheel force	Rear wheel force
	(t)	(t)	(t)	(t)	(t)
Four wheels on the ground	0	15.8	2.0	4.83	4.06
	5	15.8	2.0	8.99	2.41
	7	15.8	2.0	10.65	1.75
	8	15.8	2.0	11.48	1.42
Rear wheel off the ground	13	15.8	2.0	15.17	0

The data in the table indicate that the rim load is highest when the rear wheel is lifted off the ground.

**Figure 11:**
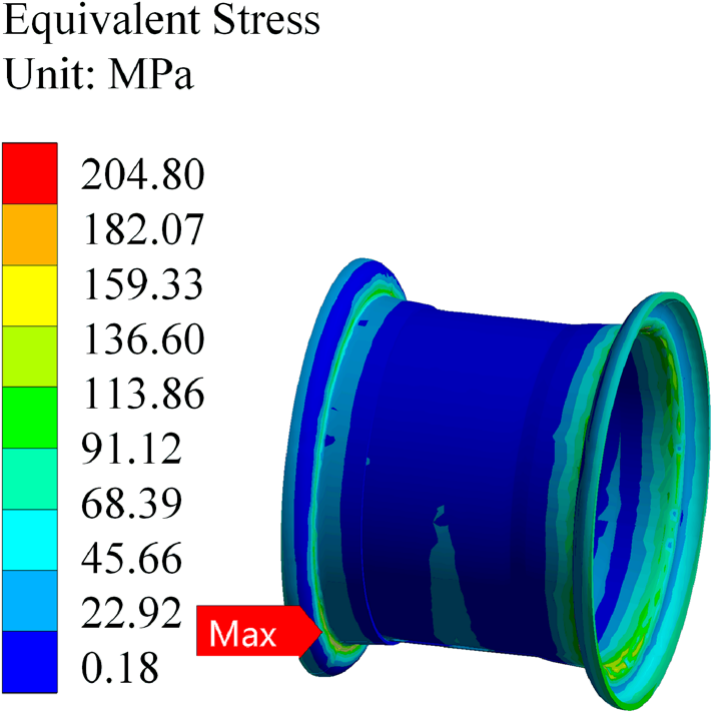
Stress in the wheel rim under a load of 13 tons.

**Figure 12:**
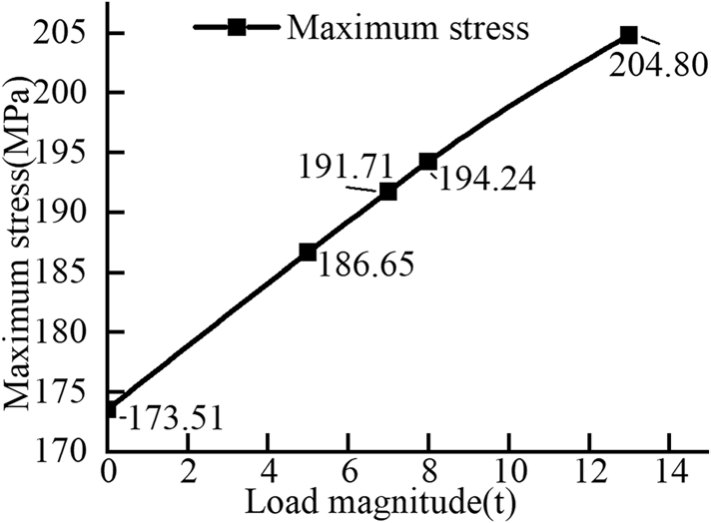
Load-maximum stress plot.

The rim material is designated as Q235, and various load magnitudes from the table are applied to the finite element simulation for calculation. The simulation results are depicted in Figs. 11 and 12. When the rim is stressed, the maximum stress and strain are located at the transition point of the outer rim fillet.

As the operation of the loader causes the position of the valve hole to rotate with respect to the rim, varying the position of the valve hole consequently impacts the maximum stress of the rim.

**Figure 13:**
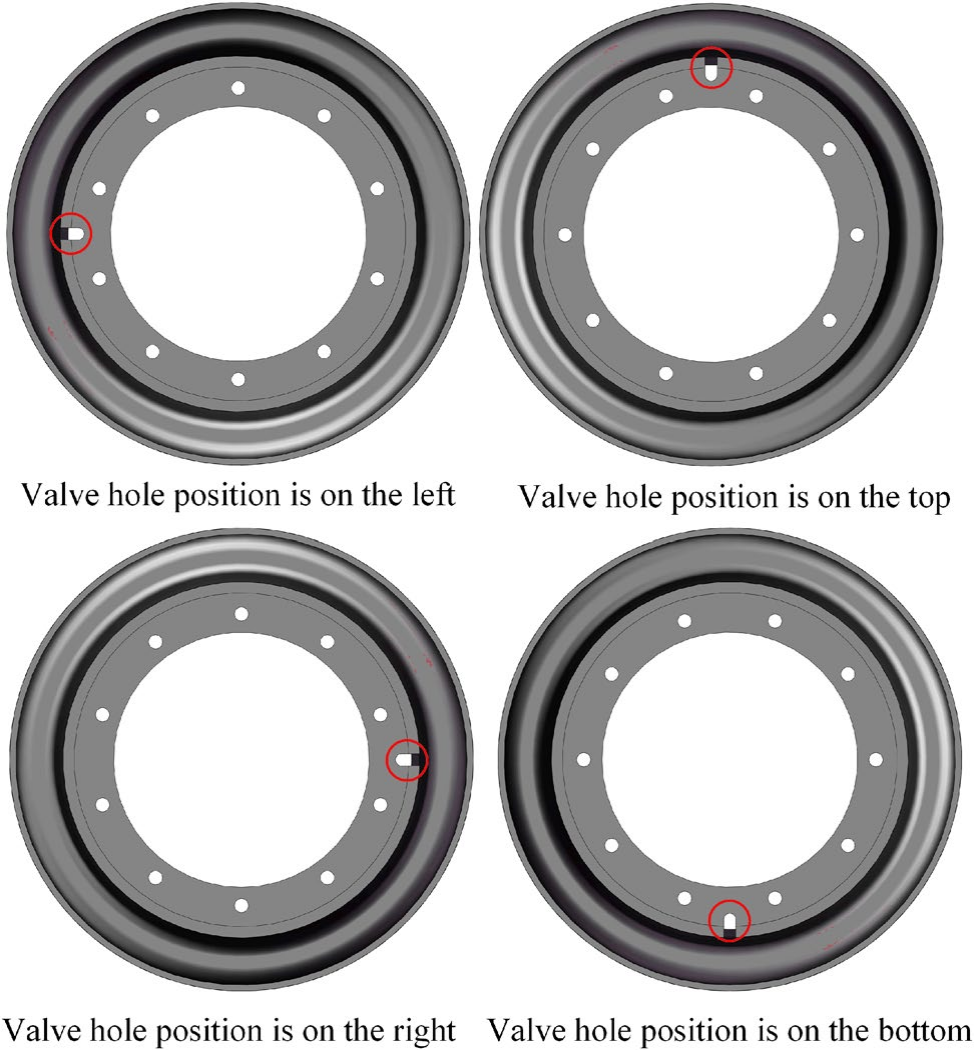
Valve hole positions on the rim.

**Figure 14:**
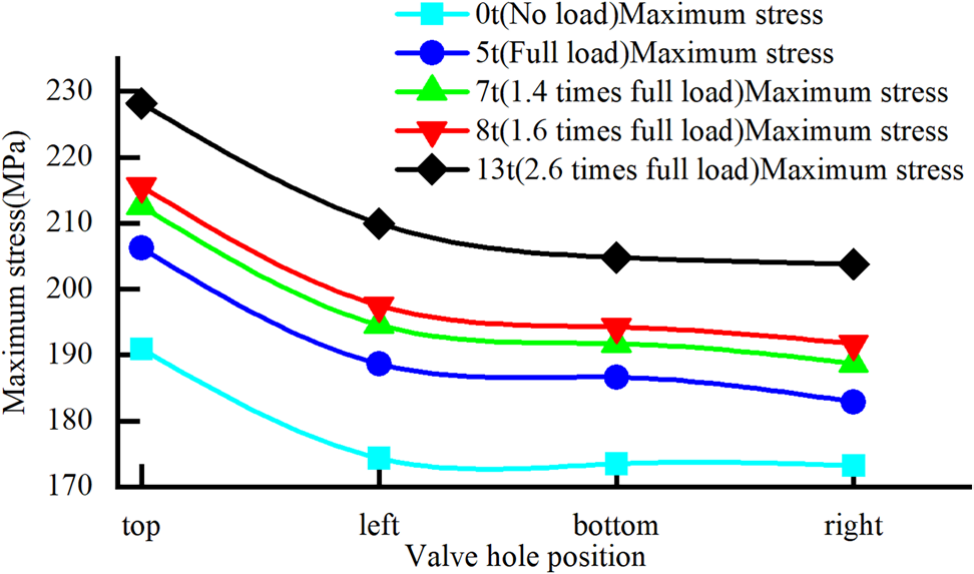
Stress trends for different valve hole positions under the same load.

We consider the Q235 material as an example to analyse the four classic positions of the valve hole: upper, lower, left, and right, as shown in Fig. 13. By applying the same load, we compare which valve hole position yields the highest stress. The calculation results are presented in Fig. 14.

From the curve in the figure, it is evident that under identical loads, the rim stress is highest when the valve hole is positioned at the top, reaching a maximum value of 228.2 MPa when the rear wheel is lifted off the ground.

## Multiobjective optimization design of various rim materials

### Design of experiments (DOE)

Following the static analysis of the valve upwards position of the hold and the lifting condition of the rear wheel under varying rim loads, a design of experiment (DOE) experiment is performed. The experimental points are extracted within the numerical range of the optimization space for the optimization parameters. Each set of experimental points represents the size of an optimization model. Subsequently, simulation operations are conducted on all experimental points, and the calculation results are fitted through mathematical statistics.

In this article, the optimal space-filling design method is employed to extract experimental points. In this instance, 2 optimization parameters are optimized, resulting in the definition of 9 groups of experimental points for calculation.

### Construction of response surfaces

Following the completion of the DOE experiment, the kriging interpolation method is employed to construct the response surface. The response surface generated by this method effectively passes through all the experimental points, ensuring high fitting accuracy. The convergence correlation error is set to 5$\%$. If the convergence requirements cannot be met, refinement points are automatically added for the refinement operation, with a maximum limit of 5. If the convergence requirements are met during the refinement process, the procedure is terminated.

**Figure 15:**
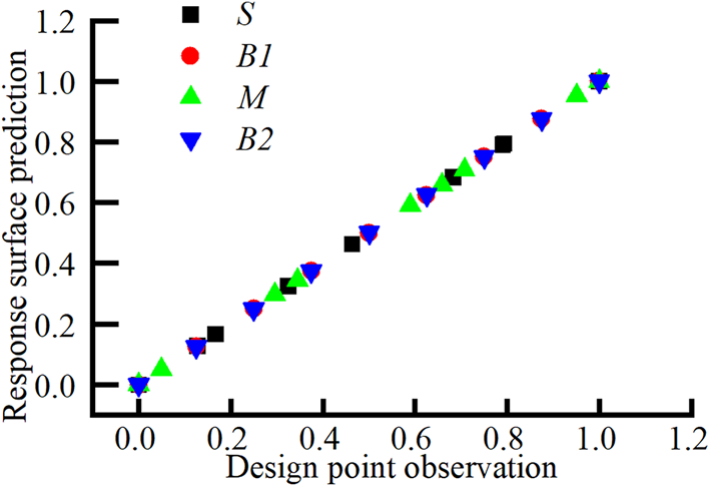
Response surface scatter plot.

Figure 15 displays the scatterplot of fitting between multiple variables. The distribution of different variables along a straight line suggests that the response surface fitted by this calculation is of high quality and suitable for multiobjective optimization.

**Figure 16:**
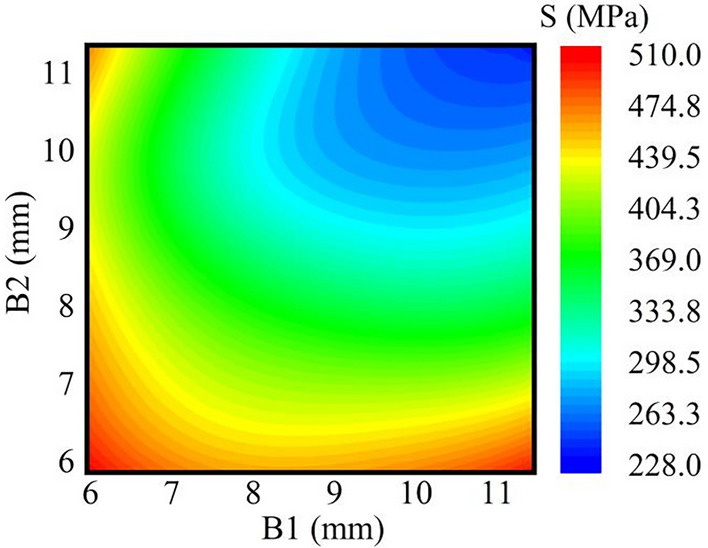
Cloud plot of the thickness-stress response.

Figure 16 displays the response surface nephogram for the rim wall thickness and maximum stress. It is evident from the figure that as the wall thicknesses B1 and B2 decrease, the maximum stress increases.

**Figure 17:**
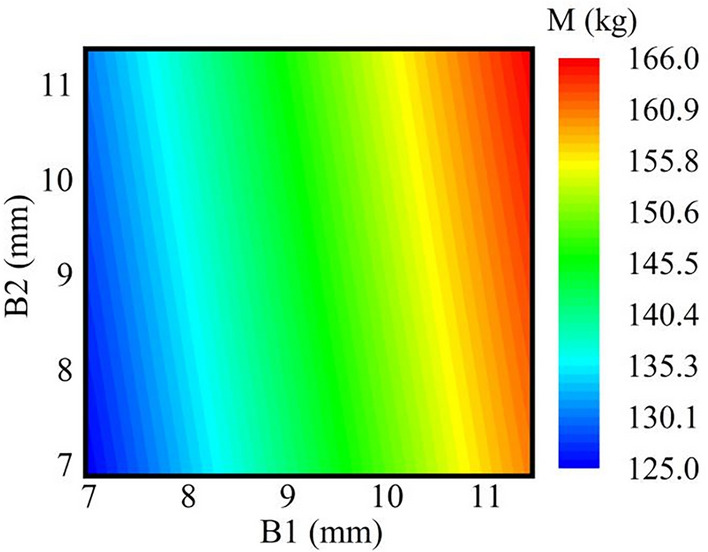
Cloud plot of the thickness-wheel rim mass response.

Figure 17 presents the response surface nephogram for wall thickness and rim mass. It is evident from the figure that the rim body size B1 has a highly significant impact on the overall quality.

### Multiobjective optimization design based on response surfaces

Using the response surface model constructed above, the MOGA algorithm is employed to iteratively optimize the optimization goal. This multiobjective genetic algorithm can achieve relatively high accuracy in the parameter space design domain. Throughout the calculation and solution process, the algorithm incorporates sample points into the response surface model for iterative calculation, ultimately obtaining an optimal solution that meets the accuracy requirements.

The objective function for the multiobjective optimization of Q235 rims is as follows:

7 $$\begin{aligned} \left\{ \begin{aligned} find min(B1) \\ min(B2) \\ min(M) \\ seeS<235 \end{aligned}\right. \end{aligned}$$

**Figure 18:**
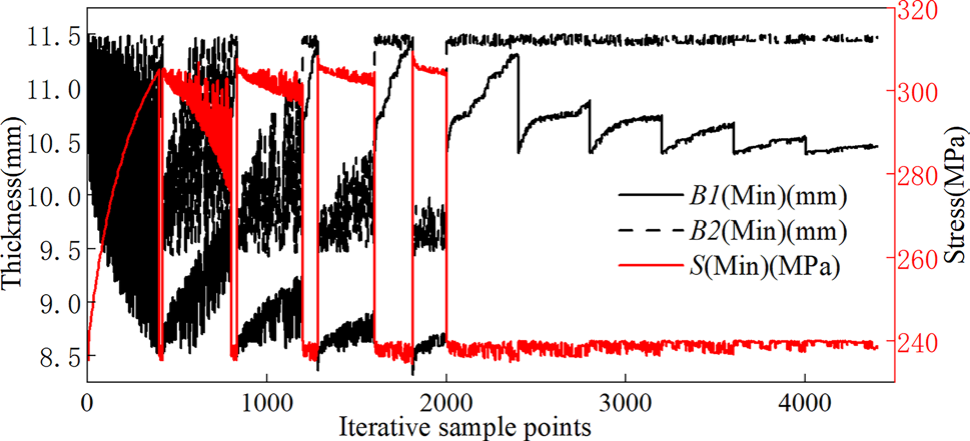
Iterative optimization plot for the Q235 material parameters.

In this calculation, the initial iteration sample points are set to 2000, with 400 iteration samples for each calculation. The maximum number of iterations is set to 20, 3 optimization candidate points are specified, and the convergence stability percentage is set to 2$\%$. The rim manufactured from Q235 achieves a stable optimization convergence of 1.57$\%$ during the optimization process, meeting the convergence requirements of multiobjective optimization. This value reaches the convergence benchmark after 11 iterative optimizations. The optimization diagram of the parameters in the multiobjective optimization process is depicted in Fig. 18. Parameters B1, B2, and S are calculated iteratively within the upper and lower limits of the optimization process shown in Table 1 until the optimal solution that meets the accuracy requirements is obtained.

The optimization results for the Q235 rim material are presented in Table 5. From the table, it is evident that this material offers limited optimization potential, with the current rim wall thickness reaching its minimum value under these operating conditions.

**Table 5:** Optimization candidate points for the Q235 material.

	Valve hole positioned upwards		
Size code	Candidate point 1	Candidate point 2	Candidate point 3
B1	11.03	11.05	11.06
B2	11.49	11.49	11.46
S	234.43	234.20	234.93
M	162.43	162.55	162.61

	Valve hole positioned downwards		
Size code	Candidate point 1	Candidate point 2	Candidate point 3
B1	10.87	10.88	10.89
B2	11.48	11.48	11.49
S	234.99	234.97	234.94
M	161.16	161.21	161.25

	Valve hole positioned on the left		
Size code	Candidate point 1	Candidate point 2	Candidate point 3
B1	10.32	10.34	10.39
B2	11.47	11.46	11.42
S	234.83	234.92	234.96
M	156.81	156.94	157.22

	Valve hole positioned on the right		
Size code	Candidate point 1	Candidate point 2	Candidate point 3
B1	11.14	11.18	11.21
B2	10.03	9.98	9.97
S	234.88	234.81	235.52
M	161.51	161.74	162.05

The wall thickness of the rim is optimized when replaced with the Q355 material. During optimization, we consider a safety factor of 1.2. Thus, the objective function for the multiobjective optimization of the rim is as follows:

8 $$\begin{aligned} \left\{ \begin{aligned} find min(B1) \\ min(B2) \\ min(M) \\ seeS<295 \end{aligned}\right. \end{aligned}$$

**Figure 19:**
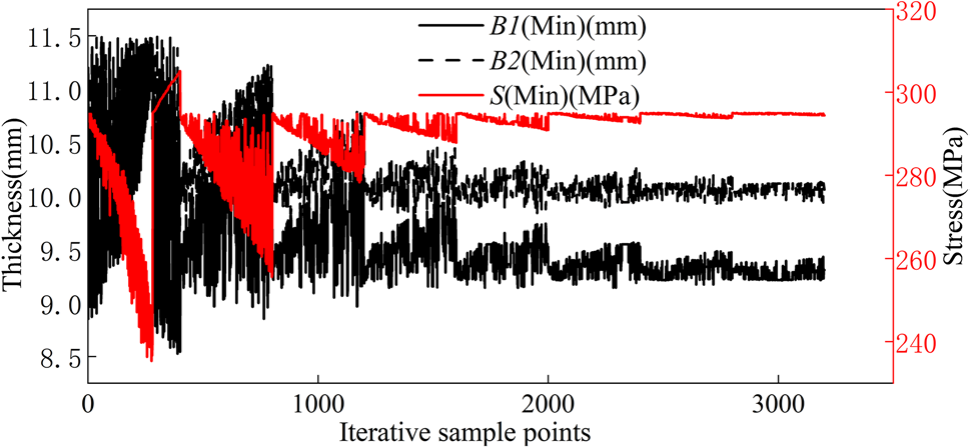
Iterative optimization plot for the Q355 material parameters.

The stable optimization convergence for the Q355 rim during the optimization process is 1.68$\%$, meeting the convergence requirements of multiobjective optimization. The rim achieves the convergence benchmark after 8 iterative optimizations. An optimization diagram of the optimization parameters in the multiobjective optimization process is shown in Fig. 19. Parameters B1, B2, and S are calculated iteratively within the upper and lower limits of the optimization process shown in Table 1 until the optimal solution that meets the accuracy requirements is obtained.

As depicted in Table 6, following the optimization of the wall thickness for the Q355 material, B1 is 10 mm, and B2 is 10 mm.

**Table 6:** Optimization candidate points for the Q355 material.

	Valve hole positioned upwards		
Size code	Candidate point 1	Candidate point 2	Candidate point 3
B1	9.54	9.52	9.58
B2	10.36	10.39	10.39
S	294.95	294.97	294.93
M	146.88	146.80	147.17

	Valve hole positioned downwards		
Size code	Candidate point 1	Candidate point 2	Candidate point 3
B1	9.28	9.32	9.22
B2	10.03	10.00	10.10
S	295.00	294.97	294.99
M	146.85	147.07	146.42

	Valve hole positioned on the left		
Size code	Candidate point 1	Candidate point 2	Candidate point 3
B1	9.50	9.48	9.53
B2	9.78	9.79	9.75
S	294.96	294.97	294.92
M	148.22	148.13	148.47

	Valve hole positioned on the right		
Size code	Candidate point 1	Candidate point 2	Candidate point 3
B1	8.69	8.71	8.75
B2	9.96	9.86	9.81
S	294.98	294.49	297.97
M	141.92	142.07	142.35

The wall thickness of the rim is optimized by replacing it with 540CL. During optimization, we consider a safety factor of 1.2. Therefore, the objective function for the multiobjective optimization of the rim is as follows:

9 $$\begin{aligned} \left\{ \begin{aligned} find min(B1) \\ min(B2) \\ min(M) \\ seeS<450 \end{aligned}\right. \end{aligned}$$

**Figure 20:**
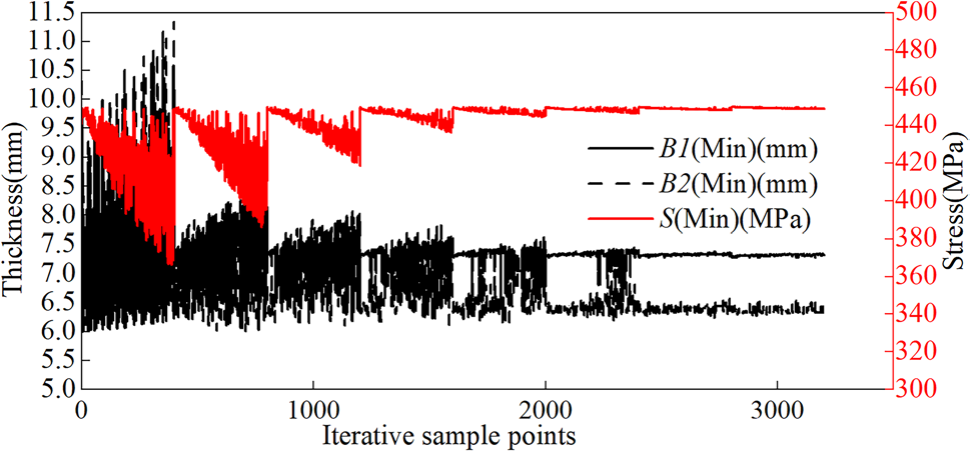
Iterative optimization plot for the 540CL material parameters.

The rim made of 540CL achieves a stable optimization convergence of 0.85$\%$ during this process, meeting the convergence requirements of multiobjective optimization. This rim reaches the convergence benchmark after 8 iterative optimizations. An optimization diagram of the parameters in the multiobjective optimization process is shown in Fig. 20. Parameters B1, B2, and S are calculated iteratively within the upper and lower limits of the optimization process shown in Table 1 until the optimal solution that meets the accuracy requirements is obtained.

As indicated in Table 7, following the optimization of the wall thickness for the 540CL material, B1 is rounded to 7 mm, and B2 is 7 mm.

**Table 7:** Optimization candidate points for 540CL material.

	Valve hole positioned upwards		
Size code	Candidate point 1	Candidate point 2	Candidate point 3
B1	7.00	7.01	7.00
B2	7.19	7.23	7.33
S	450.03	448.45	444.84
M	125.23	125.29	125.37

	Valve hole positioned downwards		
Size code	Candidate point 1	Candidate point 2	Candidate point 3
B1	6.80	6.83	6.89
B2	7.28	7.22	7.16
S	448.86	449.95	449.38
M	123.96	124.13	124.17

	Valve hole positioned on the left		
Size code	Candidate point 1	Candidate point 2	Candidate point 3
B1	6.52	6.52	6.61
B2	7.18	7.09	6.94
S	449.92	449.83	449.90
M	120.89	121.22	121.77

	Valve hole positioned on the right		
Size code	Candidate point 1	Candidate point 2	Candidate point 3
B1	6.29	6.35	6.40
B2	7.62	7.59	7.54
S	449.83	449.43	449.93
M	120.04	120.46	120.78

Based on the calculations above, two optimal sizes are ultimately determined: rims composed of Q355 material with a thickness of 10 mm and rims composed of 540CL material with a thickness of 7 mm.

## Finite element verification of the rim optimization results

### Comparison of static verification

**Figure 21:**
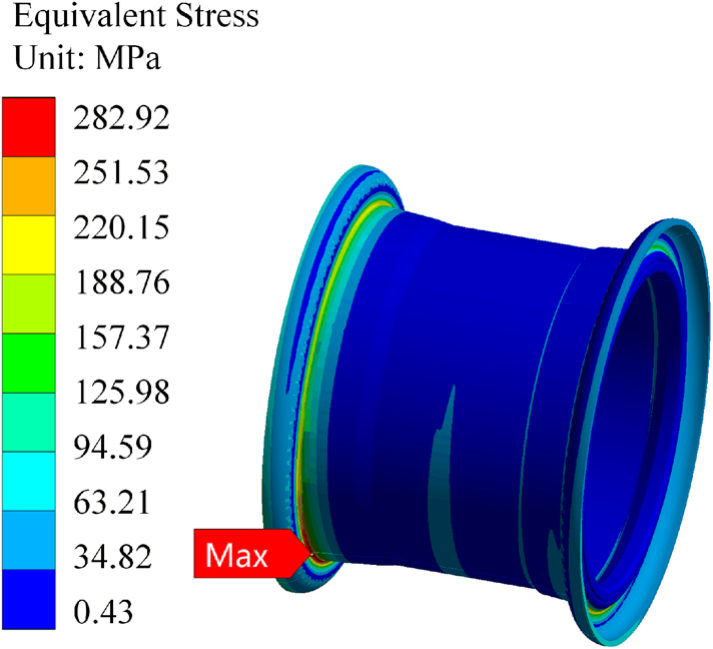
Stress cloud plot for the Q355 material.

**Figure 22:**
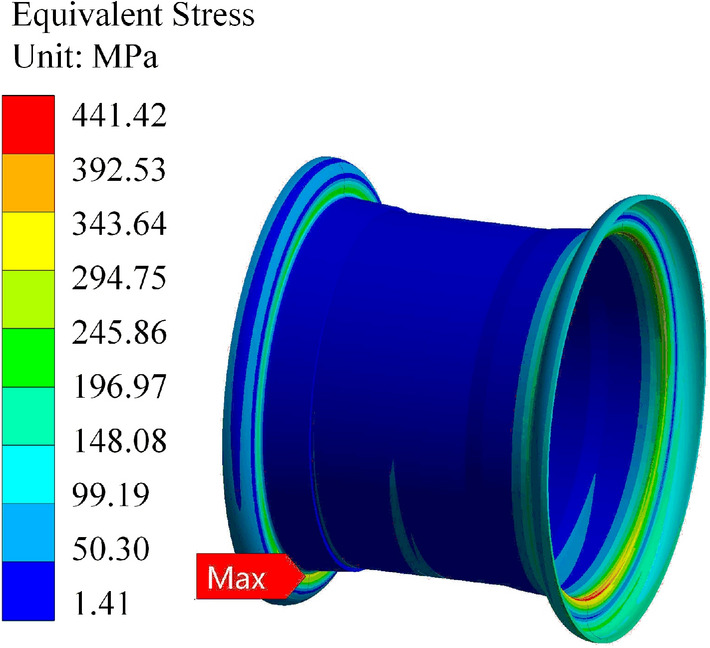
Stress cloud plot for the Q355 material.

**Figure 23:**
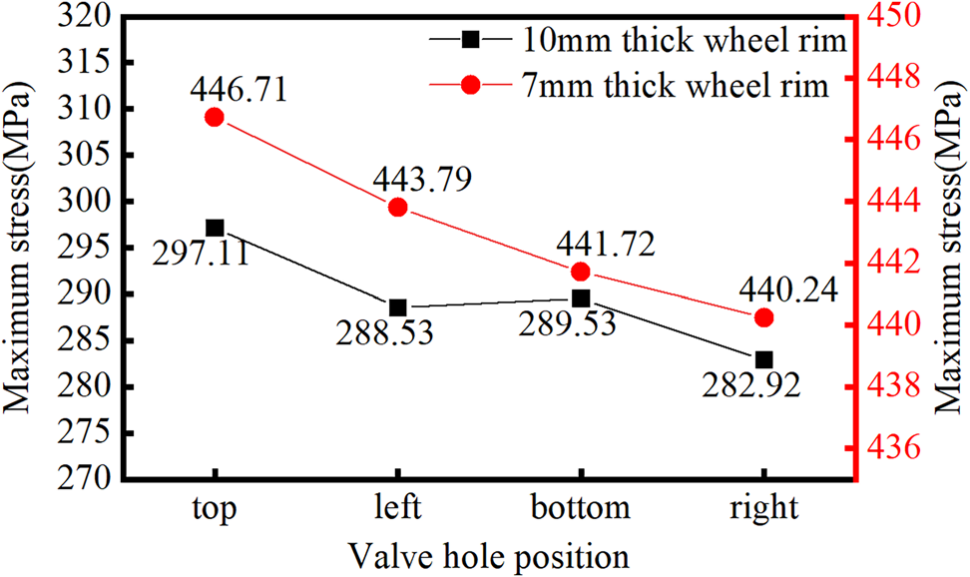
Stress results for optimized wheel rims with different valve hole positions.

Based on the static analysis data above, it is evident that the stress on the rear wheel peaks when the valve hole is positioned at the top and the rear wheel is lifted off the ground. Here, we compare the simulation data under these conditions. The finite element software uses the same boundary conditions as mentioned above for the calculations. The resulting calculation cloud diagrams are depicted in Figs. 21, 22 and 23. When the rim is stressed, the maximum stress and strain are at the transition point of the outer rim fillet.

From the figure, it is evident that the maximum stress calculation result for the Q355 material rim is 297.11 MPa, with a safety factor of 1.21. Similarly, the maximum stress calculated for the 540CL material rim is 446.71 MPa, with a safety factor of 1.19. In the calculation of the Q235 material rim mentioned above, the maximum stress is 282.2 MPa under these working conditions, with a safety factor of 1.03. From the static analysis results, it is apparent that the safety factors of the two optimization schemes are greater than those without optimization, and the 540CL rim material exhibits excellent performance.

### Comparison of radial fatigue verification

In this paper, the radial fatigue life is investigated using the nominal stress method. The nominal stress method is a common technique for analysing the fatigue lives of components and is often used in high-cycle fatigue analysis. Considering the design requirements of the wheel, it is reasonable to analyse the fatigue life of the wheel through the nominal stress method.

The S–N curves of the materials are obtained. Considering the influences of different factors, the stress curve is corrected to obtain the S–N curve of the component. Finally, the S–N curve of the component is used to calculate its life based on the theory of fatigue accumulation damage.

**Figure 24:**
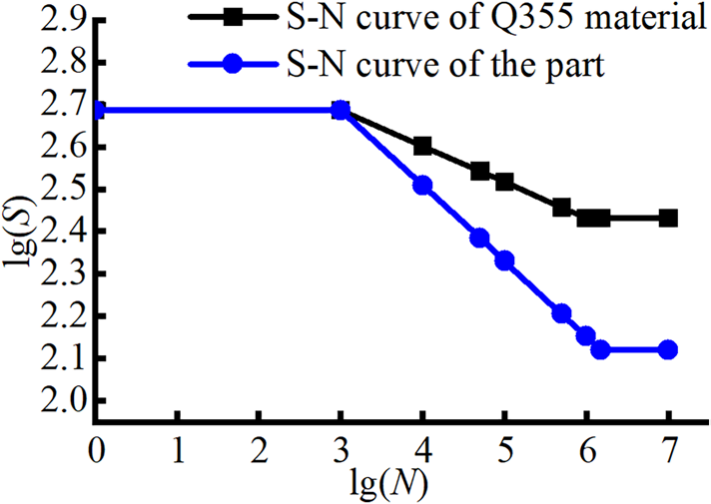
S–N curves of the Q355 material.

**Figure 25:**
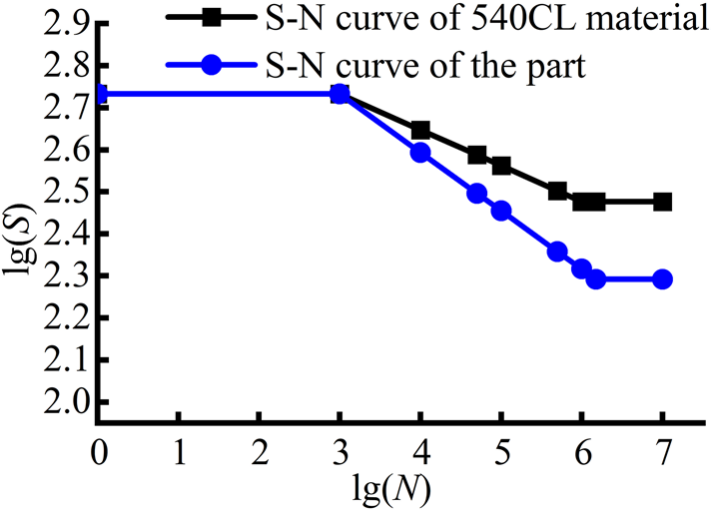
S–N curve of the 540CL material.

The S–N curves for the Q355 and 540CL materials are established based on tensile experiments to determine their tensile strengths. After correction, the S–N curves for components made from these two materials are obtained, as shown in Figs. 24 and 25.

**Figure 26:**
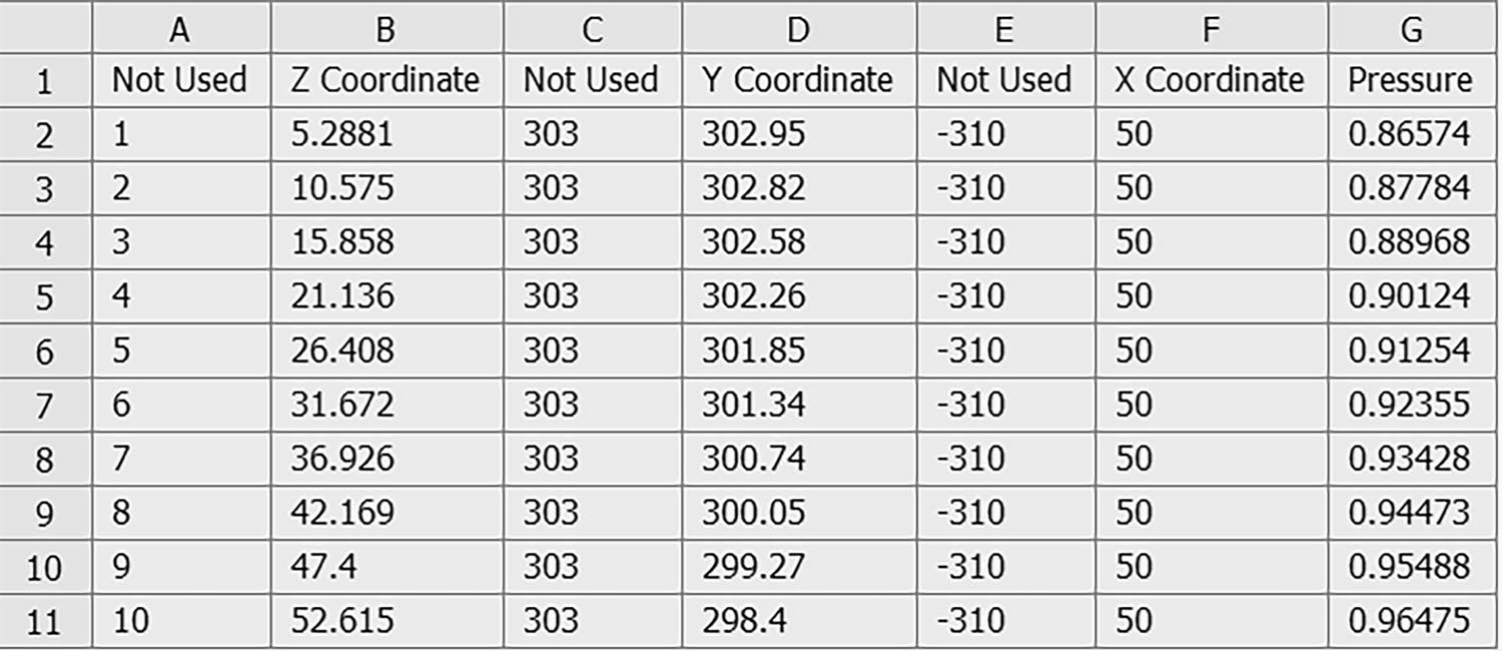
Illustration of the external data that are imported.

The S–N curves of the components are input into finite element software for fatigue life analysis by applying symmetric cyclic loads. When establishing the radial fatigue finite element simulation model, the radial load on the rim from the tire is usually simplified to a cosine load. In this paper, an external data import method is adopted, as shown in Fig. 26, where the variation in the load with respect to the angle is controlled by setting the coordinates and magnitude of the load points.

The rim data are imported into the finite element simulation software, and the following boundary conditions are applied for radial fatigue:

- The tire pressure is set to 0.4 MPa.
- Cosine load data with a magnitude of 1.114 MPa are imported.
- Fixed constraints are applied to the spoke surfaces.

**Figure 27:**
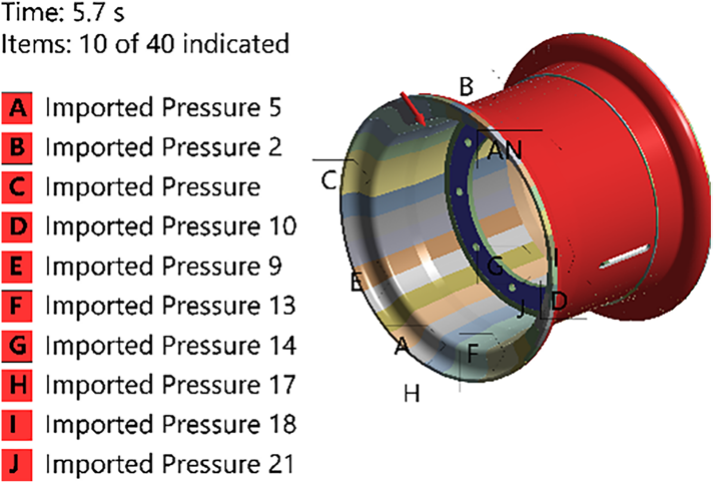
Radial fatigue loads and constraints.

**Figure 28:**
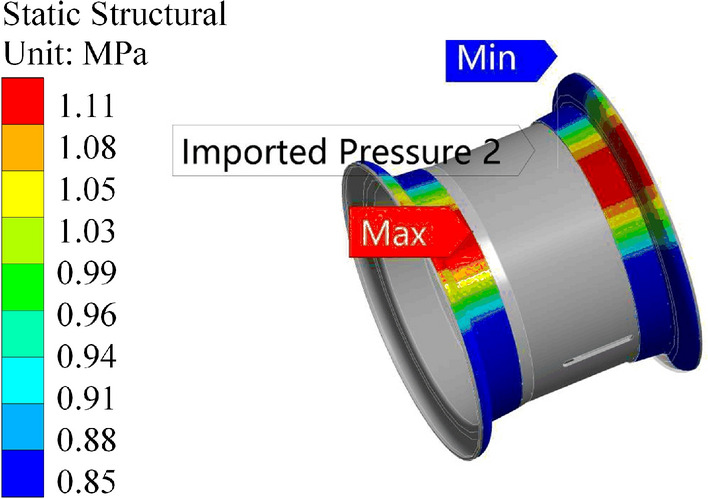
Magnitudes and distributions of the cosine force.

**Figure 29:**
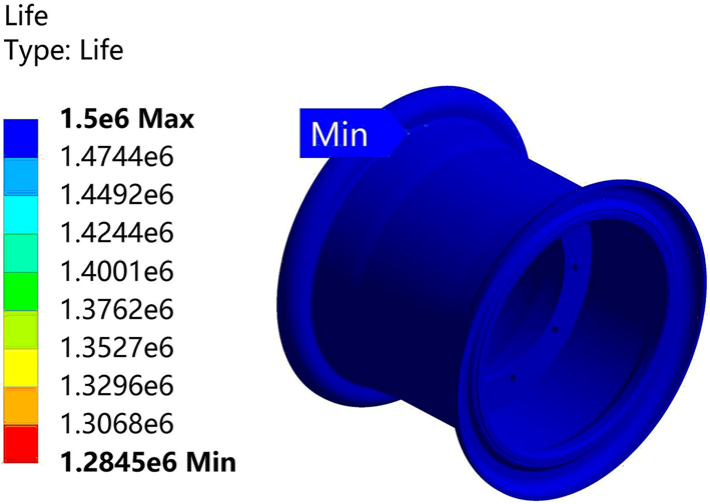
Radial fatigue life plot for the Q355 material.

**Figure 30:**
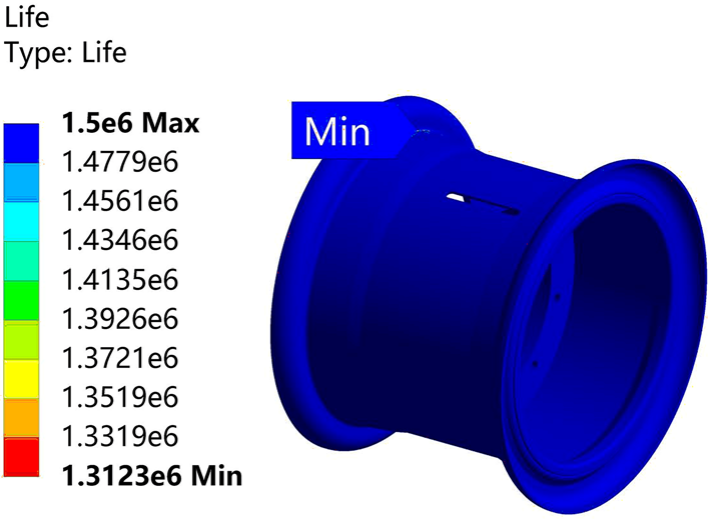
Radial fatigue life plot for the 540CL material.

The loads and boundary conditions set in the finite element software are shown in Figs. 27 and 28:

The analysis results are depicted in Figs. 29 and 30. The life of the Q355 material rim is 1.2845 million cycles, while the life of the 540CL material rim is 1.3123 million cycles, surpassing the 1 million-cycle requirement of the fatigue test. The optimized solution for the 540CL material exhibits the highest lifespan.

### Comparison of rim weight reduction

Table 8 displays the percentage of weight reduction for the rim after optimization. It is evident from the table that the weight reduction percentage for the 10 mm thick rim made of the Q355 material is 5.21$\%$. However, the weight reduction percentage for the 7-mm-thick rim made of the 540CL material is 24.78$\%$.

**Table 8:** Percentage weight reduction after wheel rim optimization.

	11.5 mm (Q235)	10 mm (Q355)	7 mm (540CL)
Weight (kg)	166.08	157.07	124.92
Weight reduction percentage ($\%$)	0	5.21	24.78

Based on the above optimization calculation and comparison, combined with the production cost and weight reduction effect of the enterprise, since the optimized rim does not change its original structural form, only the wall thickness and material are changed. Therefore, the optimized rim does not incur additional costs in terms of processing, production and assembly. Therefore, we choose the 540CL material with a thickness of 7 mm as the material with the optimal parameters.

## Verification of the optimized rear wheel test

### Optimizing rear rim production

The production of optimized rims can be divided into rim body production and spoke production. The rim body first cuts the sheet through a shearing machine and then uses a grinding wheel to remove the burrs on the sheet. After the burrs are removed, the sheet is rolled into a round shape by a rolling machine, and the two sides of the sheet are connected into one body by spot welding and wire welding. After welding, a slag blasting machine is used to remove excess welding slag from the weld, and a mould hydraulic press is used to expand the diameter of the welded sheet in accordance with the drawing requirements while ensuring ovality. After the diameter expansion is completed, one end is heated in a heating furnace and thermoformed in the mould. Finally, the inner and outer sides of the rim are machined to fulfil the surface roughness requirements.

**Figure 31:**
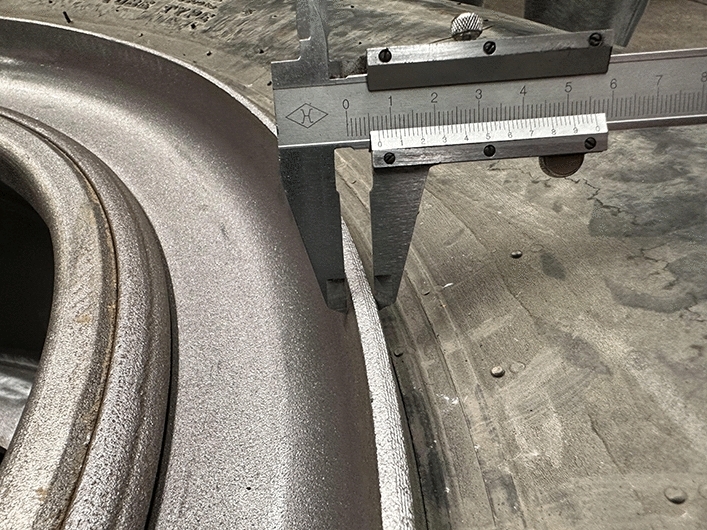
Wall thickness of the outer rim.

**Figure 32:**
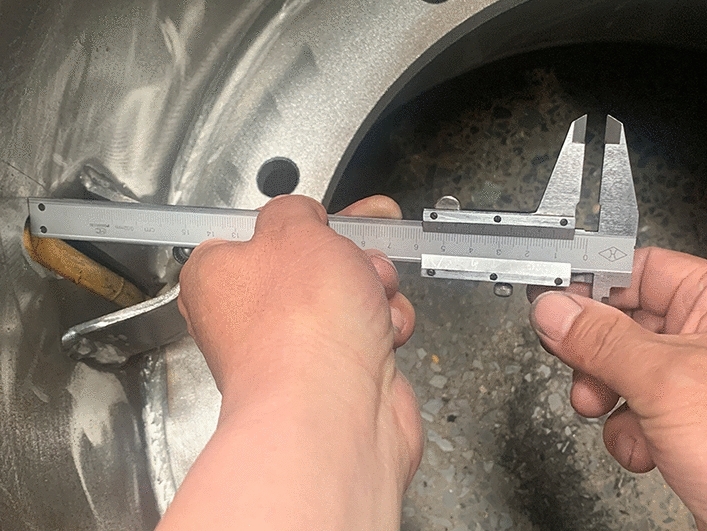
Rim body wall thickness.

To produce wheel spokes, the sheet material first needs to be plasma cut according to the size requirements of the drawing. After cutting, the inner and outer circular surfaces are machined. After machining, punching is carried out. Finally, the threaded holes on the spokes are enlarged and chamfered to meet the size requirements during rim assembly. The rim body and spokes are welded, painted, and equipped with standard locking rings and retaining rings. After the rim is produced, the rim wall thickness is first measured on site, as shown in Figs. 31 and 32. The production rim is 7 mm in thickness.

### Design of experimental plan

To verify the optimization effect and reliability of the optimized rim, it is necessary to test and verify a rim composed of 540CL with a wall thickness of 7 mm. The reliability of the finite element simulation is confirmed through a comparison of test data and simulation results.

**Figure 33:**
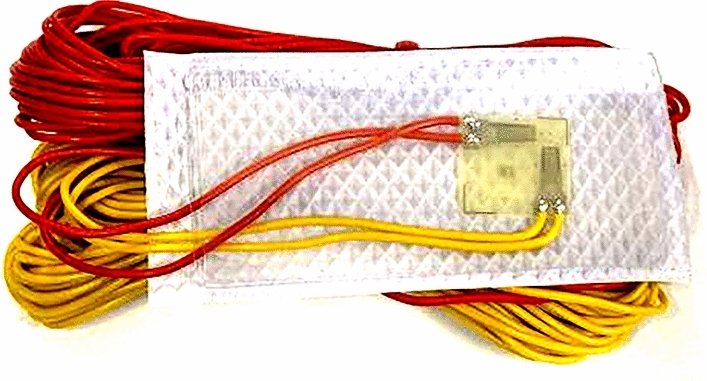
BE120-3BA right-angle strain gauge.

This test involves conducting a static stress and strain acquisition test on the wheel rim. The strain gauge collects data, which are transmitted to the strain collector through a data transmission line. Subsequently, the data are displayed in real time by connecting to a monitor. A BE120-3BA right-angle strain gauge, as depicted in Fig. 33, is employed in this test. This strain gauge, which is characterized by its compact size, ensures the accuracy of the measurement data.

**Figure 34:**
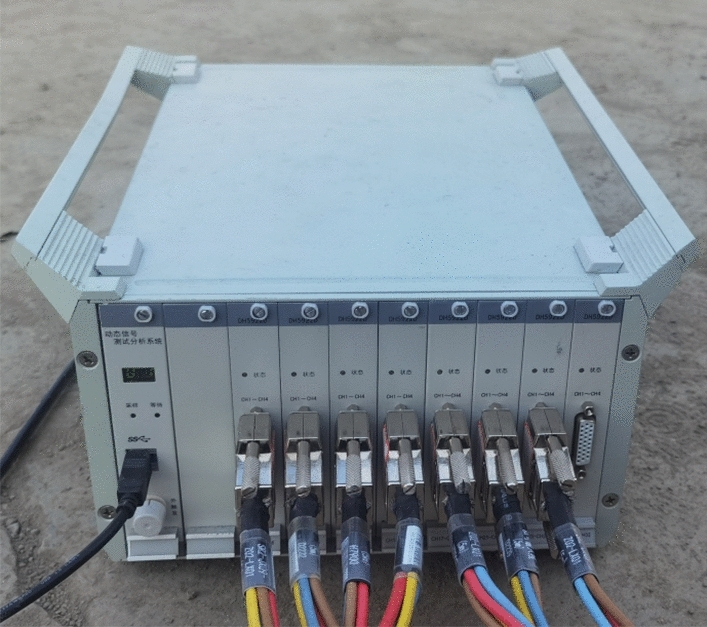
Dynamic strain gauge.

For this test, an 8-interface 32-channel strain collector is utilized to collect the static stress and strain data of the rim, as illustrated in Fig. 34.

**Figure 35:**
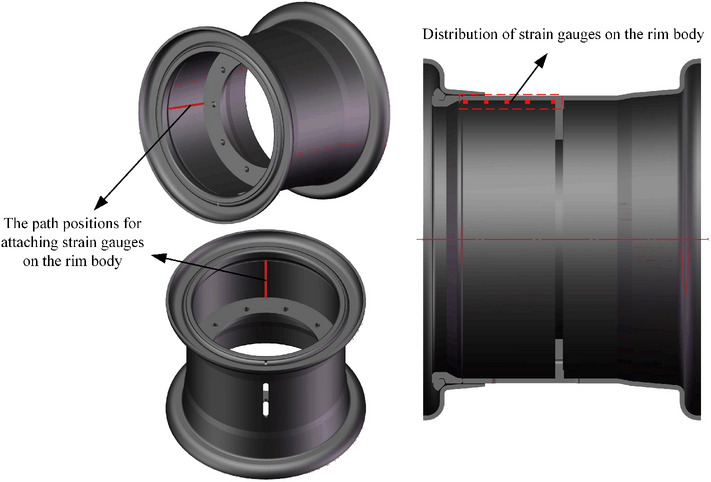
Selection of strain gauge positions on the rim.

**Figure 36:**
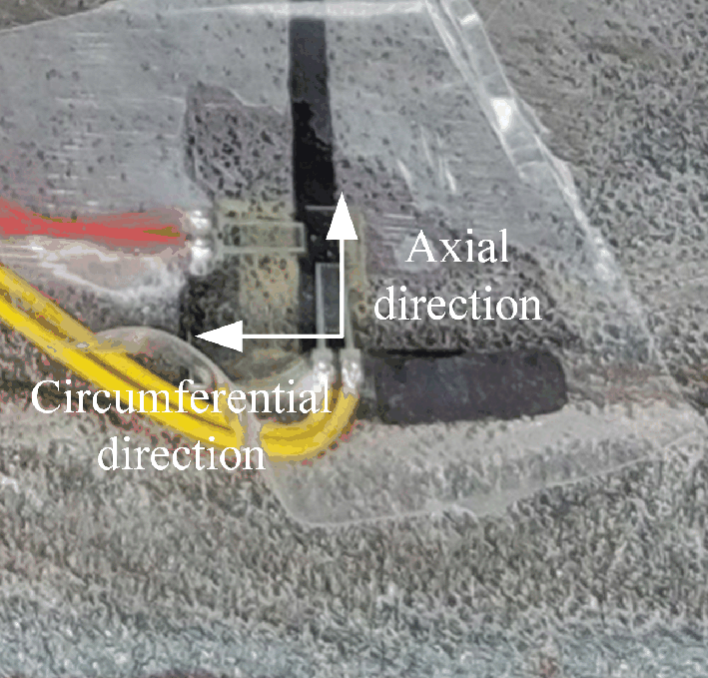
Strain gauge measuring direction.

**Figure 37:**
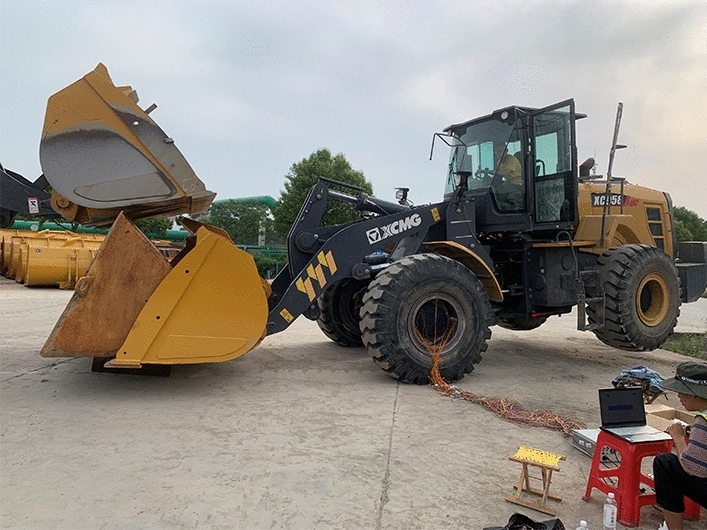
On-site conditions with the rear wheel off the ground.

When arranging the positions for the strain gauges, two paths are selected on the inner wall of the rim to the left and above the valve hole. The strain gauges are uniformly distributed along these two paths, with 5 gauges applied to each path, totalling 10 gauges. The locations where the strain gauges are applied on the rim are illustrated in Fig. 35. One section of the strain gauge on the rim body along the axial direction and one section along the circumferential direction are used to ensure the measured stress and strain direction, as shown in Fig. 36. After the pasting process is completed, it is installed on the loader for testing. The implementation site is shown in Fig. 37.

This experiment will compare the stress levels at different valve hole positions under a load of 8 t and under an overturning load. In the postprocessing of the finite element software results, the calculated results at the measurement points are extracted and compared with the experimental data.

The working condition with a load size of 8 t will be achieved by installing an 8 t weight in the loader bucket. The rear wheel lifting condition is achieved by installing an 8 t weight in the loader bucket and using another loader to apply a downwards force to the bucket to tilt the rear wheel.

### Verification and comparison of the rim body test results

The experimental data measured under different working conditions in this article are all measured when the rim is stable and stationary; thus, the strain values obtained in the experiment are taken as the measured values when they tend to be stable.

**Figure 38:**
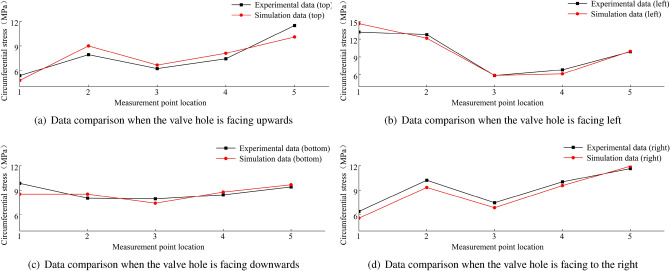
Circumferential data for the first path at an 8 t load.

**Figure 39:**
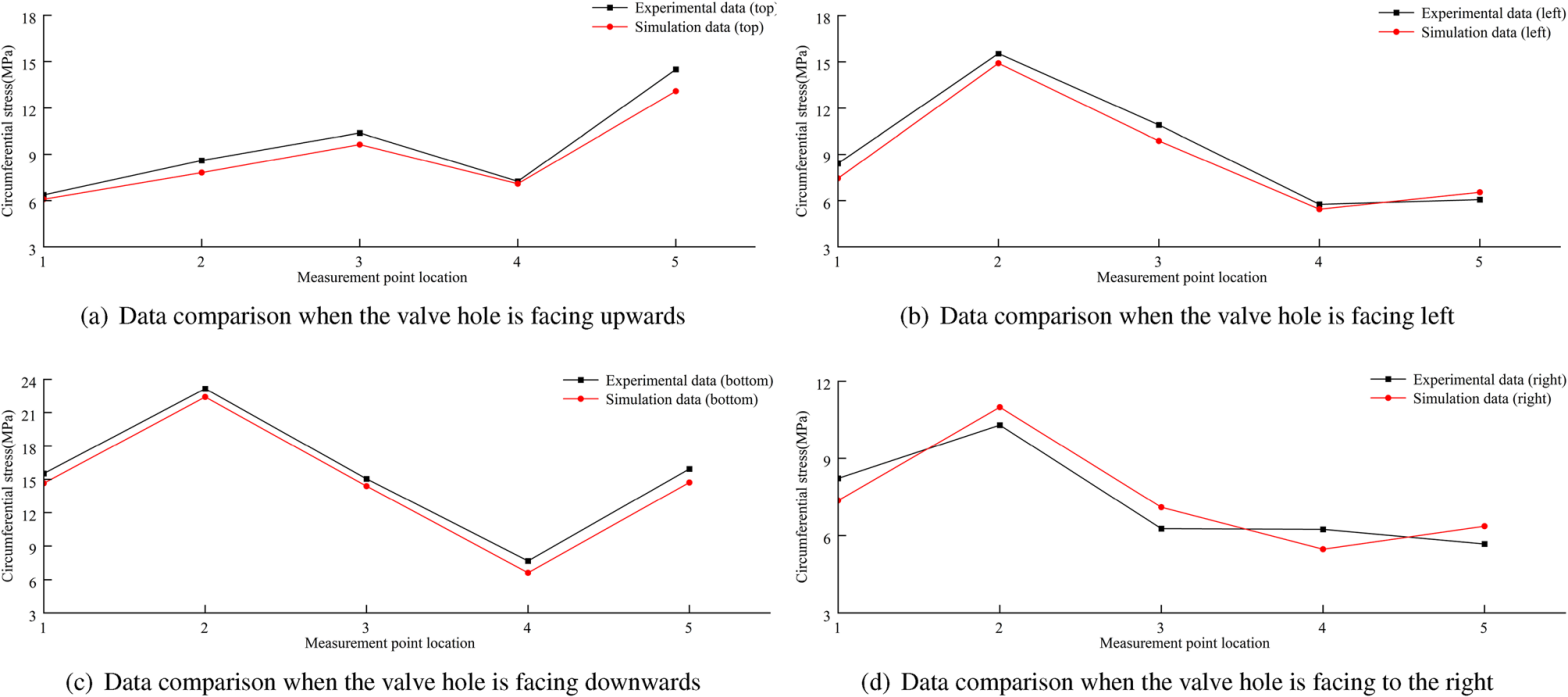
Circumferential data for the second path at an 8 t load.

**Figure 40:**
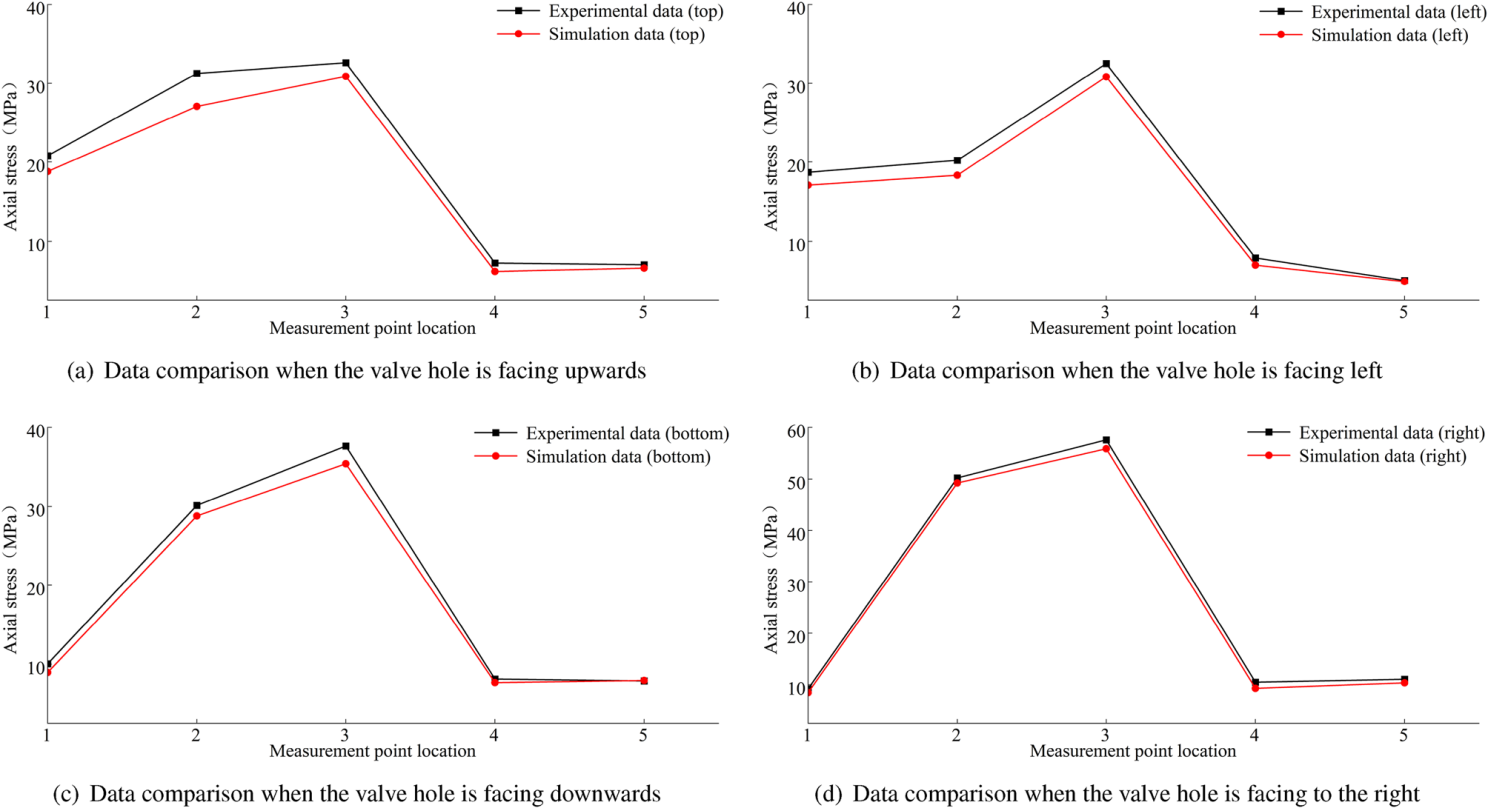
Axial data for the first path at an 8 t load.

**Figure 41:**
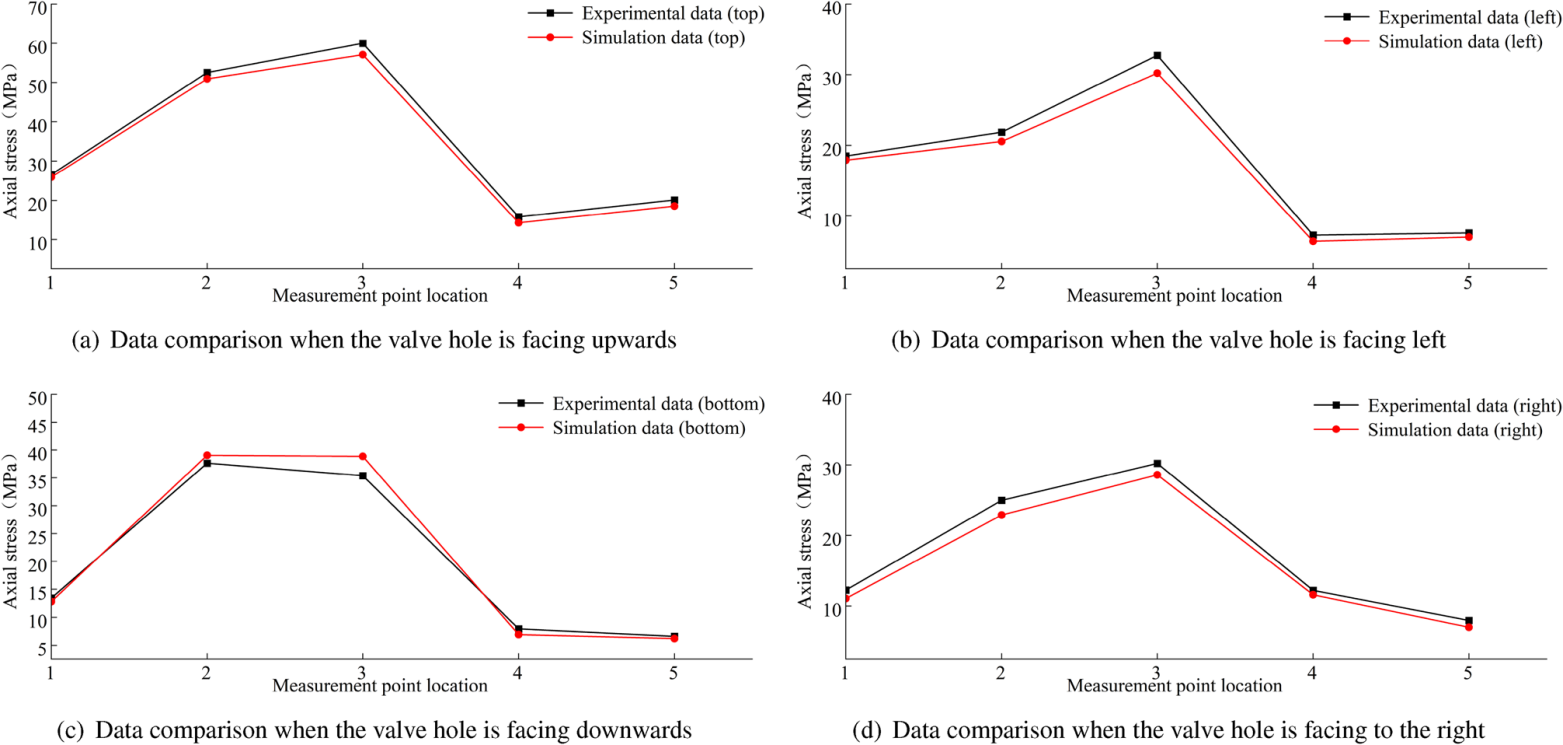
Axial data for the second path at an 8 t load.

**Figure 42:**
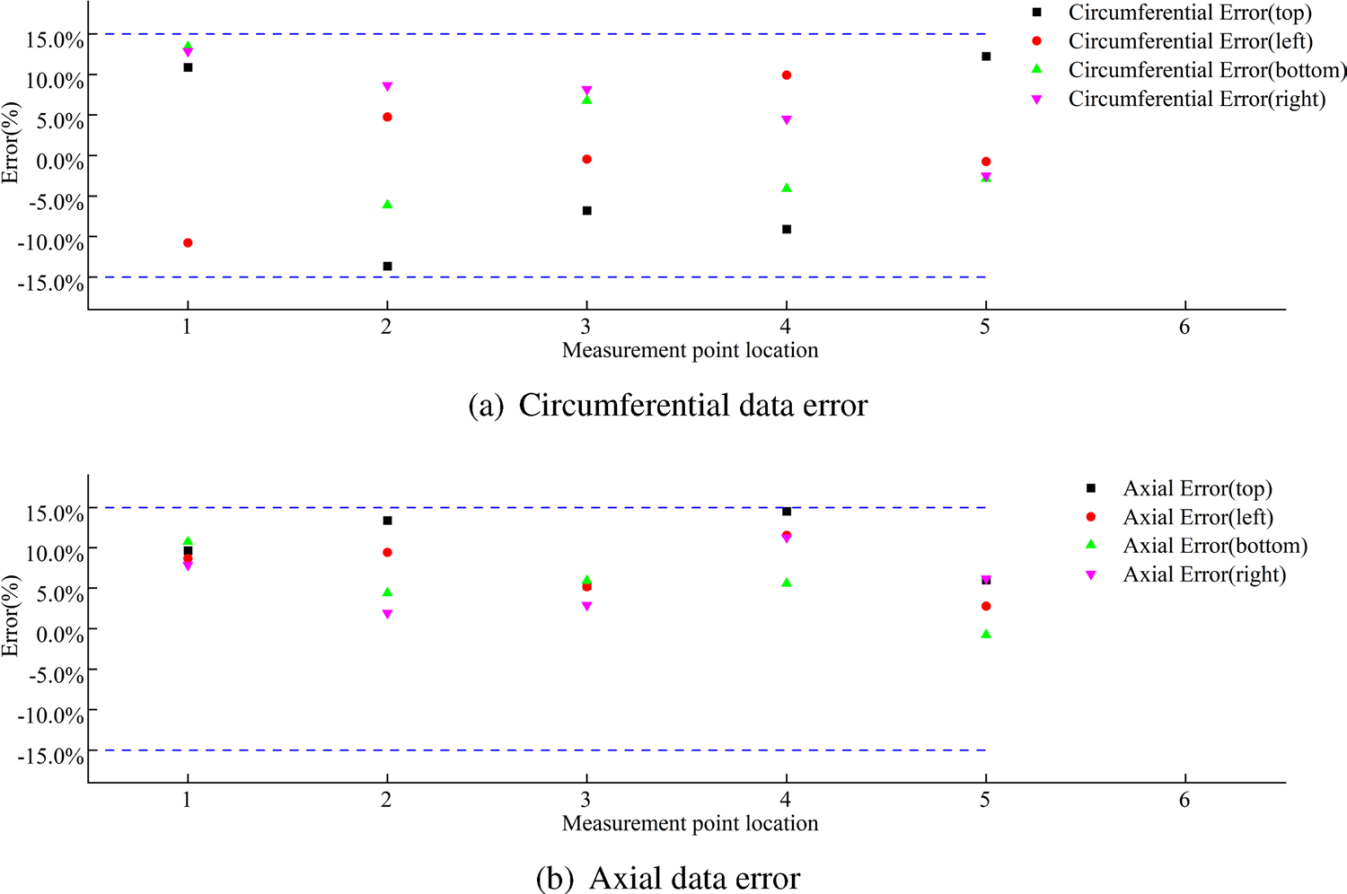
Error data for the first path at 8 t load.

**Figure 43:**
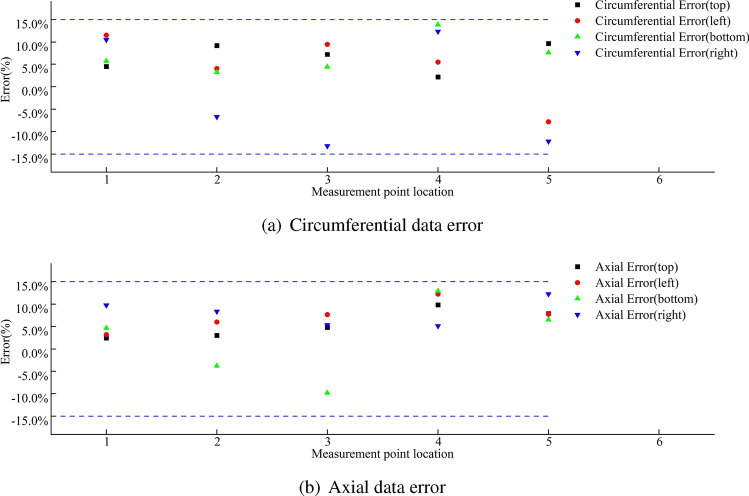
Error data for the second path at an 8 t load.

A comparison between the finite element calculations and experimental data for the stress in the wheel rim under an 8 t load is shown in Figs. 38, 39, 40 and 41. According to the figures, the simulation results follow a similar trend to the experimental data with minimal error. In the experimental data, the maximum circumferential stress is 23.15 MPa and the maximum axial stress is 60.02 MPa. The error data are shown in Figs. 42 and 43, indicating that the data errors at the measurement points are all within ± 15$\%$.

The maximum circumferential stress occurs at the first measurement point on the first path, while the maximum axial stress occurs at the third measurement point. Along the second path, the maximum circumferential stress occurs at the second measurement point, and the maximum axial stress occurs at the third measurement point.

**Figure 44:**
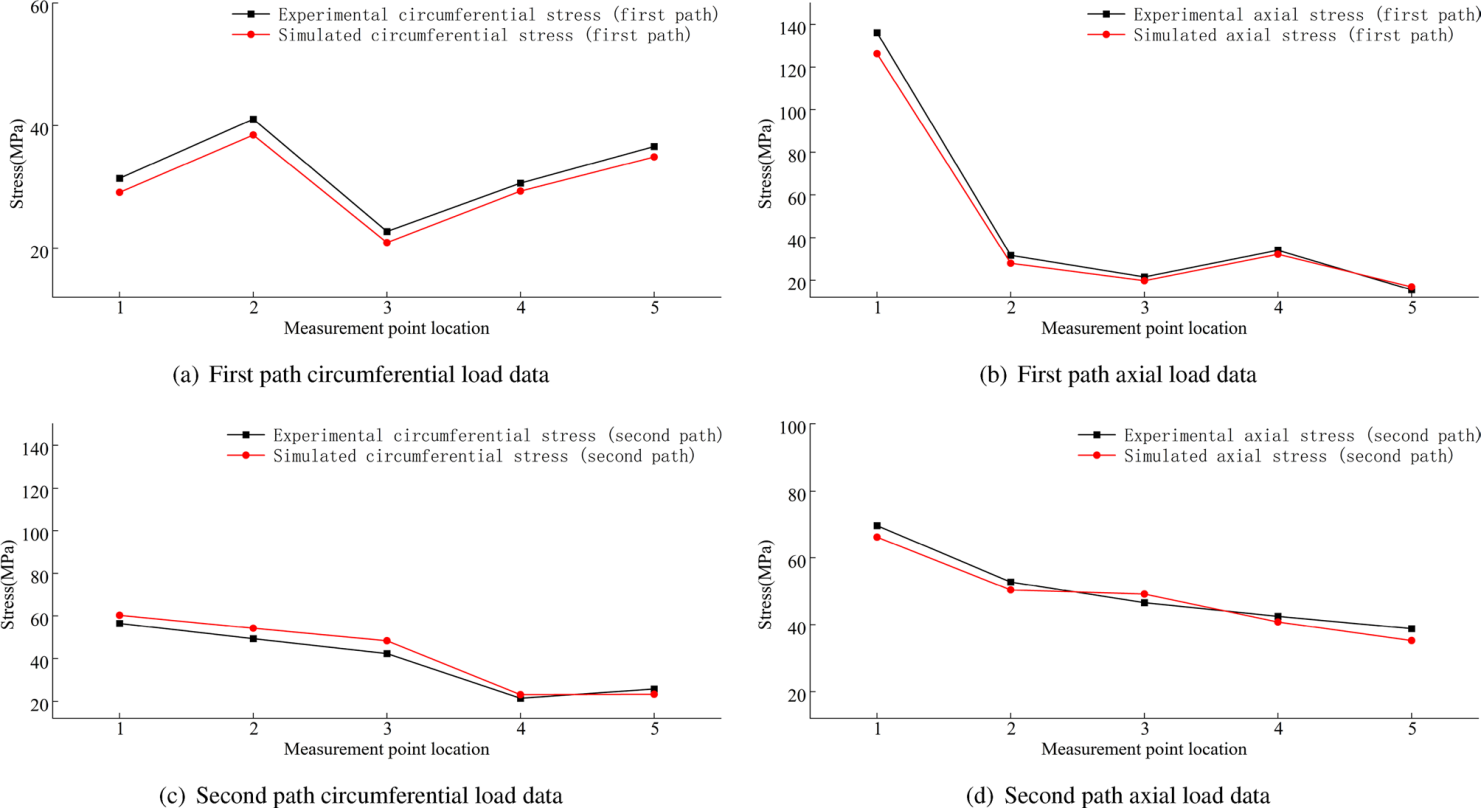
Stress data plot for the wheel rim when the rear wheel is lifted off the ground.

**Figure 45:**
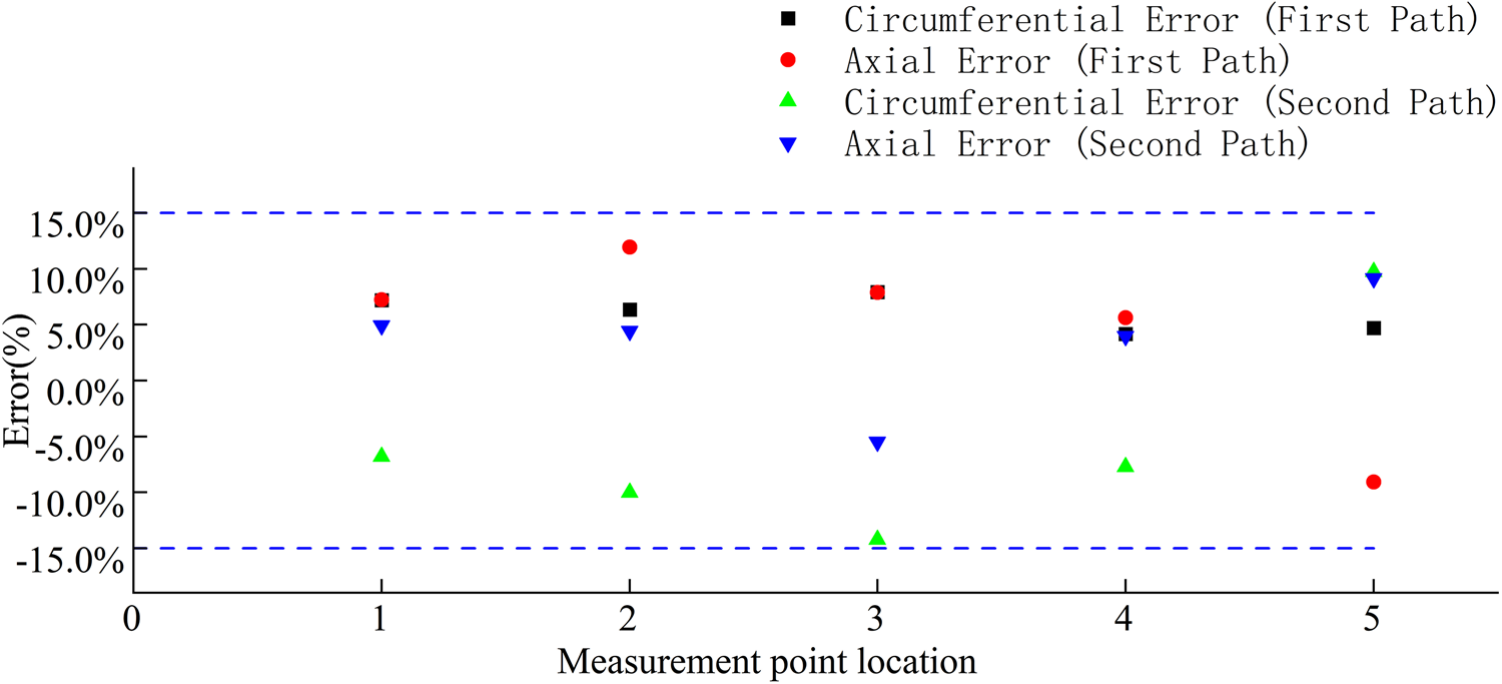
Error data plot for the wheel rim when the rear wheel is lifted off the ground.

The stress condition of the wheel rim under the condition of the rear wheel lifting off the ground is shown in Fig. 44. The figure shows that the maximum circumferential experimental stress is 56.45 MPa, and the maximum experimental axial stress is 136.16 MPa. The errors are shown in Fig. 45, and they are all within ± 15$\%$. Under this extreme condition of the rear wheel lifting off the ground, the point experiencing the maximum stress is the first measurement point near the spoke, with most experimental stress results being higher than the simulated stress results.

Summaries of the experimental and simulation data, including the error data, are provided in Tables 9, 10 and 11.

**Table 9:** Summary of the first path data when the load is 8 t.

Stress direction	Valve hole position	Measuring point location	Experimental data (MPa)	Simulation data (MPa)	Error data(%)
Circumferential stress	Top	1	5.358	4.774	10.91
		2	7.915	8.993	− 13.63
		3	6.221	6.644	− 6.8
		4	7.416	8.089	− 9.07
		5	11.497	10.09	12.24
	Left	1	13.203	14.623	− 10.75
		2	12.766	12.17	4.74
		3	5.871	5.898	− 0.45
		4	6.84	6.162	9.91
		5	9.883	9.956	− 0.74
	Bottom	1	9.851	8.529	13.42
		2	8.031	8.523	− 6.14
		3	7.955	7.418	6.75
		4	8.44	8.786	− 4.11
		5	9.412	9.682	− 2.86
	Right	1	6.397	5.574	12.86
		2	10.215	9.332	8.65
		3	7.464	6.853	8.18
		4	10.015	9.562	4.52
		5	11.645	11.935	− 2.49
Axial stress	Top	1	20.821	18.819	9.61
		2	31.269	27.079	13.4
		3	32.62	30.879	5.34
		4	7.209	6.162	14.53
		5	7.004	6.582	6.02
	Left	1	18.714	17.091	8.67
		2	20.227	18.328	9.39
		3	32.516	30.839	5.16
		4	7.859	6.949	11.58
		5	5.002	4.863	2.79
	Bottom	1	10.032	8.957	10.72
		2	30.127	28.802	4.4
		3	37.652	35.426	5.91
		4	8.134	7.681	5.57
		5	7.873	7.933	− 0.76
	Right	1	9.246	8.519	7.86
		2	50.223	49.257	1.92
		3	57.57	55.887	2.92
		4	10.514	9.33	11.26
		5	11.077	10.394	6.16

**Table 10:** Summary of the second path data when the load is 8 t.

Stress direction	Valve hole position	Measuring point location	Experimental data (MPa)	Simulation data (MPa)	Error data(%)
Circumferential stress	Top	1	6.376	6.089	0.05
		2	8.597	7.804	0.09
		3	10.378	9.631	0.07
		4	7.245	7.09	0.02
		5	14.495	13.094	0.1
	Left	1	8.412	7.443	0.12
		2	15.531	14.902	0.04
		3	10.911	9.877	0.09
		4	5.763	5.449	0.05
		5	6.07	6.543	-0.08
	Bottom	1	15.532	14.648	0.06
		2	23.154	22.415	0.03
		3	15.046	14.385	0.04
		4	7.68	6.619	0.14
		5	15.935	14.719	0.08
	Right	1	8.226	7.363	0.1
		2	10.297	10.986	-0.07
		3	6.27	7.097	-0.13
		4	6.243	5.473	0.12
		5	5.674	6.362	-0.12
Axial stress	Top	1	26.587	25.938	0.02
		2	52.538	50.949	0.03
		3	60.026	57.133	0.05
		4	15.809	14.256	0.1
		5	20.112	18.527	0.08
	Left	1	18.498	17.908	0.03
		2	21.881	20.565	0.06
		3	32.729	30.224	0.08
		4	7.308	6.416	0.12
		5	7.62	7.029	0.08
	Bottom	1	13.415	12.789	0.05
		2	37.639	39.079	-0.04
		3	35.41	38.886	-0.1
		4	7.95	6.924	0.13
		5	6.619	6.19	0.06
	Right	1	12.253	11.059	0.1
		2	25.003	22.911	0.08
		3	30.207	28.59	0.05
		4	12.229	11.602	0.05
		5	7.982	7.005	0.12

**Table 11:** Summary of rim body stress data when the rear wheel is lifted off the ground.

Path	Stress direction	Measuring point location	Experimental data (MPa)	Simulation data (MPa)	Error data(%)
First path	Circumferential stress	1	31.384	29.12	7.213
		2	41.059	38.45	6.353
		3	22.74	20.94	7.917
		4	30.592	29.31	4.19
		5	36.589	34.86	4.726
	Axial stress	1	136.163	126.28	7.258
		2	31.743	27.95	11.948
		3	21.583	19.88	7.891
		4	34.101	32.18	5.632
		5	15.51	16.92	-9.091
Second path	Circumferential stress	1	56.455	60.3	-6.81
		2	49.255	54.19	-10.02
		3	42.33	48.36	-14.245
		4	21.518	23.18	-7.725
		5	25.853	23.34	9.72
	Axial stress	1	69.619	66.18	4.94
		2	52.715	50.37	4.448
		3	46.565	49.12	− 5.488
		4	42.462	40.77	3.985
		5	38.786	35.23	9.169

The analyses indicate that test errors primarily stem from the following three aspects:

- During valve hole position adjustments for testing, reliance on visual observation and verbal instructions to the driver often leads to challenges in precisely aligning the valve hole in the four directions, causing directional deviations in the test.
- The loader inaccurately positions the standard weight in the centre of the bucket at the test site, potentially causing an eccentric load during the test and thereby introducing additional errors.
- Variations in operating skills and subjective judgements may arise during testing due to manual operation and evaluation, potentially resulting in uncertainty in the test results.

The test validates the stress conditions of the optimized rim body under various working conditions. Based on the test results presented above, the discrepancy between this test and simulation is minimal, suggesting that the finite element simulation data in this article are fairly accurate.

## Conclusions

In this paper, the rim wall thickness and material are adjusted while varying the maximum stress and mass of the rim under identical working conditions. From this process, we generate a response surface cloud diagram. Based on this diagram, the wall thicknesses of various materials are optimized via a multiobjective genetic algorithm. Ultimately, the optimization results reveal that the optimal wall thickness for the Q355 material rim is 10 mm and that for the 540CL material rim is 7 mm.

Static, radial fatigue, and weight reduction analyses are performed on the two optimization schemes. The results indicate that the maximum stresses of rims composed of both materials are below their yield strengths, with the 540CL material rim exhibiting the highest safety factor. The lifespans of both materials exceed the 1 million-cycle fatigue test requirement, with the 540CL material optimization solution exhibiting the highest durability. The weight reduction percentage of the 10-mm-thick rim made of the Q355 material is 5.21$\%$, while that of the 7-mm-thick rim made of the 540CL material is 24.78$\%$. Considering factors such as cost and performance, a 7-mm-thick rim made of 540CL is chosen as the ultimate optimization solution.

A comprehensive machine test is performed on a 7-mm-thick rim crafted from 540CL material, and the test data are juxtaposed with the finite element calculation results. The errors across all test measurement points fall within 15$\%$, confirming the dependability of the optimized rim.

This article is of certain significance for analysing the reliability levels of wheel rims under different working conditions and the multiobjective optimization of these components. It is of certain significance for further improving product quality and meeting the requirements of increasingly reduced costs.

## Data Availability

The datasets generated and analysed during the current study are not publicly available due to the signing of a confidentiality agreement with Party A but are available from the corresponding author upon reasonable request.
